# Ultra-deep sequencing data from a liquid biopsy proficiency study demonstrating analytic validity

**DOI:** 10.1038/s41597-022-01276-8

**Published:** 2022-04-13

**Authors:** Binsheng Gong, Ira W. Deveson, Timothy Mercer, Donald J. Johann, Wendell Jones, Weida Tong, Joshua Xu

**Affiliations:** 1 Division of Bioinformatics and Biostatistics, National Center for Toxicological Research, US Food and Drug Administration, Jefferson, AR 72079 USA; 2 Kinghorn Centre for Clinical Genomics, Garvan Institute of Medical Research, Sydney, NSW Australia; 3 St Vincent’s Clinical School, Faculty of Medicine, University of New South Wales, Sydney, NSW Australia; 4 Australian Institute of Bioengineering and Nanotechnology, University of Queensland, St Lucia, QLD Australia; 5 Genomics and Epigenetics Theme, Garvan Institute of Medical Research, Sydney, NSW Australia; 6 Winthrop P Rockefeller Cancer Institute, University of Arkansas for Medical Sciences, 4301 W Markham St., Little Rock, AR 72205 USA; 7 Q2 Solutions - EA Genomics, 5927 S Miami Blvd., Morrisville, NC 27560 USA

**Keywords:** Next-generation sequencing, Data publication and archiving

## Abstract

Recently we reported the accuracy and reproducibility of circulating tumor DNA (ctDNA) assays using a unique set of reference materials, associated analytical framework, and suggested best practices. With the rapid adoption of ctDNA sequencing in precision oncology, it is critical to understand the analytical validity and technical limitations of this cutting-edge and medical-practice-changing technology. The SEQC2 Oncopanel Sequencing Working Group has developed a multi-site, cross-platform study design for evaluating the analytical performance of five industry-leading ctDNA assays. The study used tailor-made reference samples at various levels of input material to assess ctDNA sequencing across 12 participating clinical and research facilities. The generated dataset encompasses multiple key variables, including a broad range of mutation frequencies, sequencing coverage depth, DNA input quantity, etc. It is the most comprehensive public-facing dataset of its kind and provides valuable insights into ultra-deep ctDNA sequencing technology. Eventually the clinical utility of ctDNA assays is required and our proficiency study and corresponding dataset are needed steps towards this goal.

## Abstract (summary)

**Table:** 

Measurement(s)	Somatic Mutation • spike-in quality control role
Technology Type(s)	Tumor Panel Sequencing
Factor Type(s)	Tumor Panel • DNA Library Input Quantity • Variant Allele Frequency
Sample Characteristic - Organism	Homo sapiens

## Background & Summary

Precision oncology utilizes next generation sequencing (NGS) of tumors for the detection of mutations that cause cancer. This can lead to more specific and individualized treatments. NGS can also be used to detect DNA shed by a tumor into the blood (aka liquid biopsy (LBx)). Our corresponding proficiency study addresses the accuracy and reproducibility of ctDNA assays using a unique set of reference materials, associated analytical frameworks, and suggested best practices. The data and methods from this study represent a seminal piece of infrastructure for future validation of liquid biopsy methods and tools.

Liquid biopsy science and clinical applications are progressing rapidly. The first liquid biopsy assay approved by the FDA in 2016 was PCR-based^1^. Four years later there were two FDA approvals for liquid biopsy tests that were NGS-based^2,3^. It is important to recognize the advantages of liquid biopsy assays versus traditional solid tumor approaches. These advantages include: i) increased safety since only a routine blood draw is required; ii) faster assay turn-around time since there is no need to schedule and coordinate a small operation or image-guided invasive procedure; iii) the potential ability to assess the heterogeneity of the malignant process, meaning assessing DNA from both the primary tumor and metastatic foci; iv) longitudinal testing prospects with time-based analyses that have not been possible before primarily due to patient safety issues; and v) new treatment monitoring strategies. For instance, blood is collected at a patient’s home for ctDNA analysis, then a medical oncologist can adjust the therapeutic plan. This can all happen outside of a traditional hospital or medical office environment.

Due to the low cell-free DNA (cfDNA) content in the circulation and low frequency of ctDNA molecules, ctDNA sequencing requires PCR amplification and ultra-deep sequencing, which introduces artifacts, PCR errors, sequencing errors, etc. Significant efforts have been made by academic and industrial scientists to overcome the challenges inherent in ctDNA sequencing. Nevertheless, the accuracy, sensitivity, and reproducibility of ctDNA assays remained unclear. The Sequencing Quality Control Phase II (SEQC2) Oncopanel Sequencing Working Group proposed a comprehensive study design, involving multiple ctDNA assays, multiple testing laboratories, multiple DNA input levels, multiple sequencing depths and other technologies, to assess the analytical performance of ctDNA assays at each major stage of the ctDNA sequencing workflow using a set of contrived reference DNA samples. Five pioneer companies, including Burning Rock Biotech, Integrated DNA Technologies, Illumina, Roche Sequencing Solutions, and Thermo Fisher Scientific, enrolled in the study to evaluate their cutting-edge ctDNA sequencing assays. The dataset generated within the study holds great value, not only for the primary goal of the original study aiming at the evaluation of the analytical validity of ctDNA sequencing assays^4^, but also for further analyses to elicit additional insights. Specifically, it can be mined for better understanding of the technology from various aspects, including but not limited to: i) making the best use of unique molecular identifiers (UMIs) for error correction; ii) better understanding the impact of sequence context / genomic regions to the low-frequency variant calling; iii) improving the accuracy of small indel calling; iv) improving the accuracy of variant calling with spike-in controls; and v) developing bioinformatics pipelines or tuning the parameters for better sensitivity and reproducibility for low-frequency variants.

The use of liquid biopsy assays to assess cancer related variants in a variety of clinical settings is increasing. Our proficiency study provides a framework for future studies, especially well-designed and powered clinical trials. Currently there is insufficient evidence of clinical validity and utility for the majority of cancer-based liquid biopsy assays, especially regarding early-stage diagnosis, treatment monitoring, and minimal residual disease monitoring. Clinical validity and utility are eventually required for liquid biopsy assays in various clinical contexts and our proficiency study and corresponding data are important and needed steps towards these goals.

## Methods

### Study design

**Fig. 1:**
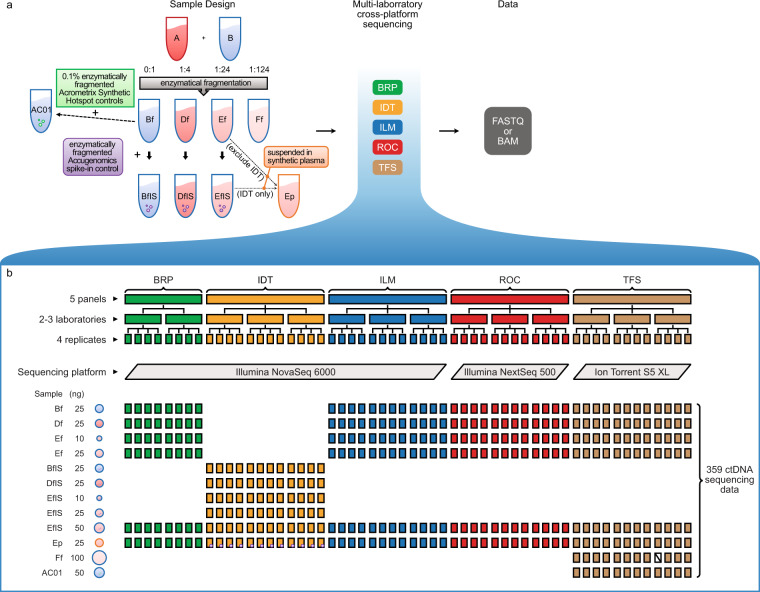
Study design. (**a**) Sample A and Sample B were mixed at ratios of 0:1, 1:4, 1:24, and 1:124, and then enzymatically fragmented to create Bf, Df, Ef, and Ff. Enzymatically fragmented Accugenomics spike-in control was added to Bf, Df, and Ef to create BfIS, DfIS, and EfIS. Sample Ef was suspended in synthetic plasma to create Ep. 0.1% enzymatically fragmented Acrometrix Synthetic Hotspot controls were added to Sample Bf to create Sample AC01. Sample Ef (Sample EfIS for panel IDT) was suspended in synthetic plasma solutions to create Sample Ep. (**b**) The illustration demonstrates the workflow of sample distribution, library preparation, and sequencing data processing. A set of reference samples were distributed to each of the testing laboratories. Four technical library replicates were prepared for each sample at each laboratory. DNA libraries were then sequenced using one of the three sequencing platforms. This figure is modified from Figure 3 in our related work^4^.

A set of contrived reference samples were created using the established Sample A, which was genotyped to have ~40,000 “known variants” and ~10.2 Mb of “known negatives” in defined coding regions (a.k.a., consensus target region (CTR)), and Sample B, which is a non-cancer background cell line^5^. Sample A and Sample B were mixed at different ratios and enzymatically fragmented to create Sample Df (a.k.a., LBx-high), Ef (a.k.a., LBx-low), and Ff, which harbor “known variants” at different variant allele frequency (VAF) levels. Enzymatically fragmented Accugenomics spike-in control^6^ and 0.1% enzymatically fragmented AcroMetrix synthetic hotspot controls^7^ were added for testing their usability. Five ctDNA assays, i.e., Burning Rock Biotech Lung Plasma v4 ctDNA assay (Burning Rock Biotech), Integrated DNA Technologies (IDT) xGen Non-small Cell Lung Cancer ctDNA assay (Integrated DNA Technologies), Illumina TruSight Tumor 170 + UMI (Illumina, Inc.), Roche AVENIO ctDNA Expanded Kit (Roche Sequencing Solutions), and Thermo Fisher Oncomine Lung Cell-Free Total Nucleic Acid Research Assay (Thermo Fisher Scientific) were used in this study. Twelve independent laboratories across the United States, United Kingdom, China, and Australia were recruited to perform the ctDNA assays. Each assay was performed at 2–3 independent testing laboratories, with four technical replicates per lab for each ctDNA sample at each input quantity. The LBx-low sample (Sample Ef or EfIS) was analyzed at three input quantity levels, while other samples were analyzed at one input quantity level. Not all reference samples at every input quantity levels were analyzed with all five ctDNA assays. A total of 359 DNA libraries were prepared and sequenced with one of the three sequencing platforms, i.e., Illumina NextSeq500, Illumina NovaSeq6000, and Thermo Fisher Ion S5 XL (Fig. 1).

### Genomic DNA libraries construction of reference samples

Sample A is composed of an equal mass pooling of 10 gDNA samples prepared from Agilent’s Universal Human Reference RNA (UHRR) cancer cell lines^8^. Over 42K small variants are known with high confidence in the defined regions of over 22 million bases^5^.

Sample B is a gDNA sample from a normal male cell line (Agilent Human Reference DNA, Male, Agilent part #: 5190-8848). Over 10M negative positions (positions absent of variation in all cell-lines) were genotyped with high confidence in the defined regions^5^.

Sample A and Sample B were mixed at ratios of 1:4, 1:24, and 1:124 to create Sample D, E, and F. In order to mimic the nature that ctDNA usually exists as small fragments, Sample B, D (a.k.a., LBx-high), E (a.k.a., LBx-low), and F were enzymatically fragmented to create Sample Bf, Df, Ef, and Ff. Enzymatically fragmented Accugenomics spike-in control^6^ was added to Bf, Df, and Ef to create another set of samples, namely BfIS, DFIS, and EfIS. 0.1% enzymatically fragmented Acrometrix Synthetic Hotspot controls were added to Sample Bf to create Sample AC01. Sample Ef was suspended in synthetic plasma solutions to create Sample Ep (a.k.a., LBx-low-plasma), except for panel IDT. And then in a second batch, Sample EfIS and the corresponding Sample Ep were made for panel IDT after the failure of the initial experiment. Details of the ctDNA sample preparation can be found in the related research manuscript^4^, while Ff and FfIS were not described in the manuscript, but the sample preparation was the same except the dilution ratio (Fig. 1a).

### Participating panels sign-up, test sites recruitment and sample distribution

Five oncopanel providers signed up to participate in this study. Each panel provider recruited 2–3 independent laboratories to perform their panels, following the panel providers’ standard operating procedure. A total of 12 testing laboratories were initially recruited. We then distributed the reference samples to each laboratory. Four DNA libraries were then made as technical replicates at each laboratory for each sample at each DNA input quantity levels. A total of 359 DNA libraries were prepared (Fig. 1b). Detailed information of the five participating oncopanels are listed in Table 1. For brevity, panel codes were used to identify the associated panels. Laboratory codes are listed in Table 1 to identify the test laboratories for each oncopanels.

**Table 1:** Detailed information for five participating ctDNA assays.

Panel code	Vendor	ctDNA assay		Laboratory code
BRP	Burning Rock Biotech	Lung Plasma v4		ST25, ST26
IDT	Integrated DNA Technologies	xGen Non-small Cell Lung Cancer		ST04, ST05, ST06
ILM	Illumina	TruSight Tumor 170 + UMI		ST10, ST23, ST29
ROC	Roche Sequencing Solutions	AVENIO ctDNA (Expanded Kit)		ST10, ST20, ST21
TFS	Thermo Fisher Scientific	Oncomine Lung cfDNA assay		ST22, ST23, ST24
**Panel code**	**Sequencing platform**	**Read length (bp)**	**Target genes**	**Reportable region (kb)**
BRP	Illumina NovaSeq 6000	2 × 151	168	226.9
IDT	Illumina NovaSeq 6000	2 × 151	24	110.1
ILM	Illumina NovaSeq 6000	2 × 151	154	501
ROC	Illumina NextSeq 500	2 × 151	77	161.7
TFS	Ion Torrent S5 XL		11	1.9

This table is expanded version of Supplementary Table 3 in our related work^4^ with “Laboratory code” added to better explain the file names.

### Experiment protocols

Basic information about the experimental procedures and wet-lab QC metrics are summarized in Table 1. These experimental protocols are expanded versions of descriptions in our related work^4^.

#### BRP: Burning Rock Biotech, Lung Plasma v4 ctDNA assay

Sample Ep was extracted using the QIAamp Circulating Nucleic Acid kit (Qiagen) according to the manufacturer’s instructions. After extraction, DNA concentration was quantified using Qubit 3.0 Fluorometer and concentration adjustment was performed following the organizers’ recommendation. The library prep and enrichment process were performed using Burning Rock HS UMI library preparation kit without modification. In brief, pre-fragmented SEQC2 DNA samples were end- repaired, UMI adapter ligated, and PCR enriched. About 1 μg of purified pre-enrichment UMI library were hybridized to LungPlasma^TM^ panel and further enriched following manufacturer’s instructions. The LungPlasma^TM^ panel is about 250 Kb in size and covers 168 human lung cancer related genes. Final DNA libraries were quantified using Qubit Fluorometer with dsDNA HS assay kit (Life Technologies, Carlsbad, CA). A LabChip GX Touch System, Agilent 2100 bioanalyzer or Agilent 4200 TapeStation D1000 ScreenTape was then performed to assess the quality and size distribution of the library. The libraries were sequenced on NovaSeq 6000 sequencer (Illumina, Inc., California, US) with 2 × 150 bp pair-end reads with unique dual index.

#### IDT: Integrated DNA Technologies, xGen Non-small Cell Lung Cancer ctDNA assay

Sample LBx-low-plasma was purified using the QIAamp® Circulating Nucleic Acid kit and quantified according to the methods described in the SEQC2 WG2 Sample Processing and Sequence Data Reporting SOP. Libraries were constructed using mock cfDNA samples in quadruplicate using the KAPA Hyper Prep Kit (Roche Sequencing Solutions) and IDT custom adapters. End repair and A-tailing were performed according to the manufacturer’s recommendations. For adapter ligation, 3 μM, 7.5 μM, and 15 μM stocks were used for 10 ng, 25 ng, and 50 ng input samples, respectively. Libraries were purified using 0.8X AMPure and amplified using unique dual index primers with 10, 9, and 8 cycles of PCR for 10 ng, 25 ng, and 50 ng input samples. Libraries were purified using 1X AMPure and quantified using Qubit. 500 ng of each library was captured with a custom NSCLC xGen Lockdown® Probe Panel (Integrated DNA Technologies) using the xGen Universal Blockers–TS Mix (Integrated DNA Technologies). After enrichment, libraries were amplified with the KAPA HiFi HotStart ReadyMix (Roche Sequencing Solutions) using 13 cycles for amplification. Post-capture libraries were purified with 1.5X AMPure, quantified, and pooled for sequencing on the Illumina NovaSeq S4.

#### ILM: Illumina, TruSight Tumor 170 + UMI

Libraries were prepared using the TruSight Tumor 170 Reference Guide, with modifications outlined in the TruSight UMI toolkit reference guide. Briefly, DNA samples, provided as enzymatically- fragmented material to mimic cfDNA, were end-repaired and A-tailed in a single reaction, followed by ligation to a universal adapter containing UMIs to uniquely tag each molecule going into the library preparation. Post-ligation clean-up was performed using Solid Phase Reversible Immobilization (SPRI) beads and then libraries were indexed using unique dual indexes by PCR. Target regions were captured using an overnight hybridization to biotinylated target-specific oligos which covers ~533 Kb of genomic targets across 154 genes, followed by capture with streptavidin magnetic beads. A second hybridization and capture reaction were performed followed by PCR amplification using the universal primers compatible with the sequencing flowcell. Libraries were quantified and manually normalized to 6 nM before being pooled in equal parts per library. Libraries were then further diluted and loaded using the Xp workflow on a NovaSeq 6000 S4 flowcell, with 6 libraries per lane on the flowcell. Sequencing was performed as 2 × 151 bp with 8 bp dual-indexed reads.

#### ROC: Roche Sequencing Solutions, AVENIO ctDNA Expanded Kit

The AVENIO ctDNA Expanded Kit (For Research Use Only; not for use in diagnostic procedures) is a hybridization-based workflow requiring only DNA, allowing the detection of single nucleotide variations (SNVs), insertions and deletions (Indels), fusions, and copy number variants (CNVs). Prior knowledge of the fusion breakpoint is not required, since the hybridization method targets whole introns of the genes of interest. In brief, the extracted cell-free DNA sample is initially ligated with adapters containing unique molecular identifiers, which allows for the deduplication of the eventual sequencing reads back to the original input molecules, significantly reducing undesired errors. After the ligation, PCR is used to universally amplify the ligated material; gene enrichment does not occur during the PCR. The sample is then incubated overnight with the gene panel, consisting of biotinylated probes designed for optimal enrichment of the genes of interest. The desired DNA-probe complexes are then captured on streptavidin beads, and after a series of washes, the samples are PCR-amplified. The final product of the workflow is enriched libraries ready for sequencing. The final sequencing libraries were sequenced using the Illumina NextSeq 500 sequencing platform. Sequencing results were analyzed by the AVENIO ctDNA Analysis Server v1.1 (for Research Use Only; not for use in diagnostic procedures).

#### TFS: Thermo Fisher Scientific, Oncomine Lung Cell-Free Total Nucleic Acid Research Assay

For samples that required nucleic acid extraction, the MagMAX™ Cell-Free Total Nucleic Acid Isolation Kit (https://www.thermofisher.com/order/catalog/product/A36716) (Cat. No. A3716) was used and extraction was carried out according to manufacturer’s instructions. Sequencing libraries were constructed according to manufacturer’s specifications found in the Oncomine™ Lung cfTNA Assay User Guide (https://www.thermofisher.com/order/catalog/product/A35864). Included in the user guide is the protocol for constructing and templating sequencing libraries using the Ion Chef™ Instrument (Cat. No. 4484177). Subsequently, each library was loaded on to an Ion 530™ chip & Ion 530™ Kit – Chef (Cat. Nos. A27757, A30010) which was then loaded on to Ion S5™ XL (Cat No. A27214) next generation sequencing system. Each sequencing library has a sample- specific Tag Sequencing barcode (Tag Sequencing Barcode Set 1–24, Cat. No. A31830) attached to each amplicon to enable identification of an individual sample which has been pooled with other multiplexed samples loaded on an Ion 530™ chip.

### Sequencing, data processing, and collection

The libraries from the same panel were sequenced on one of the three sequencing platforms chosen by the panel providers, including Illumina NextSeq 500, Illumina NovaSeq 6000, and one ThermoFisher IonTorrent S5 XL. Each library was sequenced only on one of the platforms, so the comparison across sequencing platforms of the exact same DNA library is not available with the dataset. Sequencing data was required to be shared within the SEQC2 Oncopanel Working Group via Illumina BaseSpace Sequence Hub or by uploading data to the sFTP server hosted at Stanford University. Either FASTQ or BAM format was used for data sharing. All the data was collected at the National Center for Toxicological Research (NCTR), organized, and renamed in a consistent manner. The data was then submitted to the National Center for Biotechnology Information (NCBI) Sequence Read Archive (SRA) data repository. The data became publicly available upon the publication of the related research manuscript^4^.

### Bioinformatics pipelines and variant calling results

In this study, we considered each recommended or in-house pipeline to be part of the solution of variant detection along with the according oncopanel. In the related research manuscript, the variant calling results were reported by the panel providers’ recommended or in-house pipelines for the associated panels. The reproducibility, sensitivity, and false positive rate of the participating oncopanels were reported in the related research manuscript^4^. This data descriptor is focusing on describe the raw sequencing data for possible reuse, however, if researchers want to compare their variant calling results with the results of the recommended or in-house pipelines, the results presented in our related manuscript^4^ can be downloaded from figshare^9^ in VCF format.

## Data Records

The data have been deposited to NCBI SRA with accession number SRP296025^10^. Sample information can be found in Table 2. There are 359 NCBI SRA records in total for this study, and the total download size is 4.11 Tb. Some Sample Ff data for panel TFS were not available due to an unrecoverable data loss.

**Table 2:** Data Records at NCBI.

Sample code	Sample name	NCBI BioSample ID	SRA ID
Bf	Sample_Bf_ctDNA	SAMN16786373	SRS7834557
AC01	Sample_AC01_ctDNA	SAMN17007051	SRS7836005
BfIS	Sample_BfIS_ctDNA	SAMN17013275	SRS7841495
Df	Sample_Df_ctDNA	SAMN16786374	SRS7834558
DfIS	Sample_DfIS_ctDNA	SAMN17013276	SRS7841496
Ef	Sample_Ef_ctDNA	SAMN16786375	SRS7834559
EfIS	Sample_EfIS_ctDNA	SAMN17005115	SRS7834560
Ep	Sample_Ep_ctDNA	SAMN17005116	SRS7834561
Ff	Sample_Ff_ctDNA	SAMN17007052	SRS7836006

A detailed list of all 359 NCBI SRA records can be found in Online-only Table 1. The “library_ID” column shows the individual DNA library identifier, which combines the sample ID, panel code, testing laboratory code, DNA input amount, and library replicate together with “_”. There is a number “2” right after the panel code to distinguish this presenting liquid biopsy study from the oncopanel study^11^ under the SEQC2 umbrella project, which uses “1” as identifier, using the same panel codes. The “filetype” column show the file type of the according NCBI SRA record, where “fastq” indicates the FASTQ format and “bam” indicated the BAM format. Data for panel ROC is provided in unmapped BAM format. Data for TFS is provided in mapped BAM format, and the reference genome used for reads mapping is listed in the “Reference Genome” column. Data for the other three panels is provided in FASTQ format. Details of read processing for all panels and read mapping for panel TFS can be found in Code Availability. Several filenames may be listed in columns “filename” and “filename2”. These filenames are the original filenames used when uploading the data files to NCBI SRA. If you retrieve the data using NCBI’s SRA ToolKit, you may have different filenames, which may be related to the SRR accession number. Filenames start with “library_ID”, followed by DNA input quantity, then, replicate ID, the read identifier, and filename extension. If the file type is “bam”, there is one file listed for each record for TFS and two files (for two different deduplicated methods) for ROC, ending with “.bam” as the filename extension. If the file type is “fastq”, the files end with “.fastq.gz”. You will find pairs of files listed for each record, where “R1” and “R2” refer to the left and right reads.

## Technical Validation

The dataset is self-validated by design through multiple aspects, (a) each of the reference samples was library- prepared and sequenced 4 times at one testing laboratory, which is added to at least 8 times (2 or 3 testing laboratories per oncopanel) for each oncopanel, and high intra- and cross-lab reproducibility was observed;^4^ (b) Sample Ef were tested on three different DNA input quantity levels; (c) Two spike-in control products were used for quality control.

## Usage Notes

This manuscript reflects the views of the authors and does not necessarily reflect those of the U.S. Food and Drug Administration. Any mention of commercial products is for clarification only and is not intended as approval, endorsement, or recommendation.

Variant calling should be restricted to the designed regions of each panel and BED files for the five panels can be downloaded from figshare^12^. For panel ILM and ROC, blocklist of genomic regions have been excluded, and for panel TFS, a list of pre-defined hotspot alleles representing variants are provided.

Because enzymatically fragmented Accugenomics spike-in control was added to create BfIS, DFIS, and EfIS, the raw reads of these datasets should map against both the native template (NT) of reference genome (GRCh37/hg19 or GRCh38/hg38) and the modified internal standard (IS) genome template to split NT and IS reads, as stated in our related work^6^.

Sample AC01 was created with Sample B and enzymatically fragmented Acrometrix Synthetic Hotspot controls^7^, which introduces 500 + somatic variants that are frequently identified in cancer. The list is provided in VCF format for both hg19 and hg38 and can be downloaded from figshare^13^.

## Code availability

Data were provided in FASTQ format (for panel BRP, IDT and ILM) or unmapped BAM format (for panel ROC) if the DNA libraries were sequenced with Illumina sequencing platforms, and data were provided in mapped BAM format (for panel TFS) if the DNA libraries were sequenced with Thermo Fisher Scientific’s Ion Torrent system, processed and mapped against hg19 with Torrent Suite Software (https://github.com/iontorrent/TS). The details including the data processing flow, software and parameters for all panels as well as read mapping for panel TFS are expanded versions of descriptions in our related work^4^.

### BRP read processing

After demultiplex and moving 6-bp UMI to the sequence header using bcl2fastq^14^ v2.20 (Illumina), sequence data in FASTQ format were filtered using the Trimmomatic^15^ 0.36 with parameters “HEADCROP:2 SLIDINGWINDOW:8:20 MINLEN:50”. (HEADCROP: Cut the specified number of bases from the start of the read).

### IDT read processing

IDT libraries were prepared with IDT custom adapters which contain 3-bp degenerate unique molecular identifiers (UMIs). Picard (http://broadinstitute.github.io/picard/) v2.18.9 IlluminaBasecallsToSam was used to demultiplex BCL files and generate unmapped bam files. The in-line UMIs were extracted using fgbio (https://github.com/fulcrumgenomics/fgbio) v0.7.0 ExtractUmisFromBam with the read structure 3M2S146T 3M2S146T, and–molecular-index-tags = ZA ZB,–single-tag = RX parameters. Illumina adapters sequences were marked using Picard v2.18.9 MarkIlluminaAdapters and trimmed in Picard v2.18.9 SamToFastq when generating FASTQ files for alignment.

### ILM read processing

The libraries prepared from enzymatically- fragmented DNA were processed using Illumina’s standard internal pipeline with a few modifications to remove artefactual- variants that were present at low levels in Sample B. Briefly, reads were demultiplexed, trimmed of adaptors, and converted into the FASTQ format using bcl2fastq^14^ v2.20.

### ROC read processing

The AVENIO ctDNA analysis pipeline is commercially available in the Oncology Analysis Server v1.1 (for Research Use Only; not for use in diagnostic procedures). It reads lane-level (BCL format) data from a run of plasma samples on a NextSeq 500, converts to raw sequence data by Illumina’s bcl2fastq^14^ v2.20 program, and then demultiplexes the data per sample using the specific sample adapter sequence ligated during its sample prep step.

### TFS read processing

Signal processing and base calling were performed using Torrent Suite Software (https://github.com/iontorrent/TS) v5.8 using default parameters for the Oncomine TagSeq Liquid Biopsy. The signal processing step involves of modeling the pH dynamics on the semiconductor surface taking account of the varying local pH in each individual sensor coming from the different reagent- flows across the chip and from any nucleotide incorporation that may be happening over each sensor. The base calling step consists of taking the estimated levels of nucleotide incorporation for each read and each nucleotide flow, and modeling the de-phasing process whereby some templates within each clonally amplified population run ahead or behind in terms of their nucleotide incorporation. During the base calling process, sample-specific barcodes and 3’-adapters are annotated. Once sequencing was complete, within Torrent Suite Software, resulting sequencing reads are mapped to the hg19 build of the human genome. Subsequently, consensus reads are built by binning read sets with common molecular tags.

## Online-only Table

**Online-only Table 1:** Metadata of 359 NCBI SRA records .

panel	biosample_accession	library_ID	library_layout	instrument_model	reference_genome	filetype	filename	filename2
BRP	SAMN16786373	SampleBf_BRP2_ST25_25ng_LIB1	paired	Illumina NovaSeq 6000		fastq	SampleBf_BRP2_ST25_25ng_LIB1.R1.fastq.gz	SampleBf_BRP2_ST25_25ng_LIB1.R2.fastq.gz
BRP	SAMN16786373	SampleBf_BRP2_ST25_25ng_LIB2	paired	Illumina NovaSeq 6000		fastq	SampleBf_BRP2_ST25_25ng_LIB2.R1.fastq.gz	SampleBf_BRP2_ST25_25ng_LIB2.R2.fastq.gz
BRP	SAMN16786373	SampleBf_BRP2_ST25_25ng_LIB3	paired	Illumina NovaSeq 6000		fastq	SampleBf_BRP2_ST25_25ng_LIB3.R1.fastq.gz	SampleBf_BRP2_ST25_25ng_LIB3.R2.fastq.gz
BRP	SAMN16786373	SampleBf_BRP2_ST25_25ng_LIB4	paired	Illumina NovaSeq 6000		fastq	SampleBf_BRP2_ST25_25ng_LIB4.R1.fastq.gz	SampleBf_BRP2_ST25_25ng_LIB4.R2.fastq.gz
BRP	SAMN16786373	SampleBf_BRP2_ST26_25ng_LIB1	paired	Illumina NovaSeq 6000		fastq	SampleBf_BRP2_ST26_25ng_LIB1.R1.fastq.gz	SampleBf_BRP2_ST26_25ng_LIB1.R2.fastq.gz
BRP	SAMN16786373	SampleBf_BRP2_ST26_25ng_LIB2	paired	Illumina NovaSeq 6000		fastq	SampleBf_BRP2_ST26_25ng_LIB2.R1.fastq.gz	SampleBf_BRP2_ST26_25ng_LIB2.R2.fastq.gz
BRP	SAMN16786373	SampleBf_BRP2_ST26_25ng_LIB3	paired	Illumina NovaSeq 6000		fastq	SampleBf_BRP2_ST26_25ng_LIB3.R1.fastq.gz	SampleBf_BRP2_ST26_25ng_LIB3.R2.fastq.gz
BRP	SAMN16786373	SampleBf_BRP2_ST26_25ng_LIB4	paired	Illumina NovaSeq 6000		fastq	SampleBf_BRP2_ST26_25ng_LIB4.R1.fastq.gz	SampleBf_BRP2_ST26_25ng_LIB4.R2.fastq.gz
BRP	SAMN16786374	SampleDf_BRP2_ST25_25ng_LIB1	paired	Illumina NovaSeq 6000		fastq	SampleDf_BRP2_ST25_25ng_LIB1.R1.fastq.gz	SampleDf_BRP2_ST25_25ng_LIB1.R2.fastq.gz
BRP	SAMN16786374	SampleDf_BRP2_ST25_25ng_LIB2	paired	Illumina NovaSeq 6000		fastq	SampleDf_BRP2_ST25_25ng_LIB2.R1.fastq.gz	SampleDf_BRP2_ST25_25ng_LIB2.R2.fastq.gz
BRP	SAMN16786374	SampleDf_BRP2_ST25_25ng_LIB3	paired	Illumina NovaSeq 6000		fastq	SampleDf_BRP2_ST25_25ng_LIB3.R1.fastq.gz	SampleDf_BRP2_ST25_25ng_LIB3.R2.fastq.gz
BRP	SAMN16786374	SampleDf_BRP2_ST25_25ng_LIB4	paired	Illumina NovaSeq 6000		fastq	SampleDf_BRP2_ST25_25ng_LIB4.R1.fastq.gz	SampleDf_BRP2_ST25_25ng_LIB4.R2.fastq.gz
BRP	SAMN16786374	SampleDf_BRP2_ST26_25ng_LIB1	paired	Illumina NovaSeq 6000		fastq	SampleDf_BRP2_ST26_25ng_LIB1.R1.fastq.gz	SampleDf_BRP2_ST26_25ng_LIB1.R2.fastq.gz
BRP	SAMN16786374	SampleDf_BRP2_ST26_25ng_LIB2	paired	Illumina NovaSeq 6000		fastq	SampleDf_BRP2_ST26_25ng_LIB2.R1.fastq.gz	SampleDf_BRP2_ST26_25ng_LIB2.R2.fastq.gz
BRP	SAMN16786374	SampleDf_BRP2_ST26_25ng_LIB3	paired	Illumina NovaSeq 6000		fastq	SampleDf_BRP2_ST26_25ng_LIB3.R1.fastq.gz	SampleDf_BRP2_ST26_25ng_LIB3.R2.fastq.gz
BRP	SAMN16786374	SampleDf_BRP2_ST26_25ng_LIB4	paired	Illumina NovaSeq 6000		fastq	SampleDf_BRP2_ST26_25ng_LIB4.R1.fastq.gz	SampleDf_BRP2_ST26_25ng_LIB4.R2.fastq.gz
BRP	SAMN16786375	SampleEf_BRP2_ST25_10ng_LIB1	paired	Illumina NovaSeq 6000		fastq	SampleEf_BRP2_ST25_10ng_LIB1.R1.fastq.gz	SampleEf_BRP2_ST25_10ng_LIB1.R2.fastq.gz
BRP	SAMN16786375	SampleEf_BRP2_ST25_10ng_LIB2	paired	Illumina NovaSeq 6000		fastq	SampleEf_BRP2_ST25_10ng_LIB2.R1.fastq.gz	SampleEf_BRP2_ST25_10ng_LIB2.R2.fastq.gz
BRP	SAMN16786375	SampleEf_BRP2_ST25_10ng_LIB3	paired	Illumina NovaSeq 6000		fastq	SampleEf_BRP2_ST25_10ng_LIB3.R1.fastq.gz	SampleEf_BRP2_ST25_10ng_LIB3.R2.fastq.gz
BRP	SAMN16786375	SampleEf_BRP2_ST25_10ng_LIB4	paired	Illumina NovaSeq 6000		fastq	SampleEf_BRP2_ST25_10ng_LIB4.R1.fastq.gz	SampleEf_BRP2_ST25_10ng_LIB4.R2.fastq.gz
BRP	SAMN16786375	SampleEf_BRP2_ST25_25ng_LIB1	paired	Illumina NovaSeq 6000		fastq	SampleEf_BRP2_ST25_25ng_LIB1.R1.fastq.gz	SampleEf_BRP2_ST25_25ng_LIB1.R2.fastq.gz
BRP	SAMN16786375	SampleEf_BRP2_ST25_25ng_LIB2	paired	Illumina NovaSeq 6000		fastq	SampleEf_BRP2_ST25_25ng_LIB2.R1.fastq.gz	SampleEf_BRP2_ST25_25ng_LIB2.R2.fastq.gz
BRP	SAMN16786375	SampleEf_BRP2_ST25_25ng_LIB3	paired	Illumina NovaSeq 6000		fastq	SampleEf_BRP2_ST25_25ng_LIB3.R1.fastq.gz	SampleEf_BRP2_ST25_25ng_LIB3.R2.fastq.gz
BRP	SAMN16786375	SampleEf_BRP2_ST25_25ng_LIB4	paired	Illumina NovaSeq 6000		fastq	SampleEf_BRP2_ST25_25ng_LIB4.R1.fastq.gz	SampleEf_BRP2_ST25_25ng_LIB4.R2.fastq.gz
BRP	SAMN16786375	SampleEf_BRP2_ST26_10ng_LIB1	paired	Illumina NovaSeq 6000		fastq	SampleEf_BRP2_ST26_10ng_LIB1.R1.fastq.gz	SampleEf_BRP2_ST26_10ng_LIB1.R2.fastq.gz
BRP	SAMN16786375	SampleEf_BRP2_ST26_10ng_LIB2	paired	Illumina NovaSeq 6000		fastq	SampleEf_BRP2_ST26_10ng_LIB2.R1.fastq.gz	SampleEf_BRP2_ST26_10ng_LIB2.R2.fastq.gz
BRP	SAMN16786375	SampleEf_BRP2_ST26_10ng_LIB3	paired	Illumina NovaSeq 6000		fastq	SampleEf_BRP2_ST26_10ng_LIB3.R1.fastq.gz	SampleEf_BRP2_ST26_10ng_LIB3.R2.fastq.gz
BRP	SAMN16786375	SampleEf_BRP2_ST26_10ng_LIB4	paired	Illumina NovaSeq 6000		fastq	SampleEf_BRP2_ST26_10ng_LIB4.R1.fastq.gz	SampleEf_BRP2_ST26_10ng_LIB4.R2.fastq.gz
BRP	SAMN16786375	SampleEf_BRP2_ST26_25ng_LIB1	paired	Illumina NovaSeq 6000		fastq	SampleEf_BRP2_ST26_25ng_LIB1.R1.fastq.gz	SampleEf_BRP2_ST26_25ng_LIB1.R2.fastq.gz
BRP	SAMN16786375	SampleEf_BRP2_ST26_25ng_LIB2	paired	Illumina NovaSeq 6000		fastq	SampleEf_BRP2_ST26_25ng_LIB2.R1.fastq.gz	SampleEf_BRP2_ST26_25ng_LIB2.R2.fastq.gz
BRP	SAMN16786375	SampleEf_BRP2_ST26_25ng_LIB3	paired	Illumina NovaSeq 6000		fastq	SampleEf_BRP2_ST26_25ng_LIB3.R1.fastq.gz	SampleEf_BRP2_ST26_25ng_LIB3.R2.fastq.gz
BRP	SAMN16786375	SampleEf_BRP2_ST26_25ng_LIB4	paired	Illumina NovaSeq 6000		fastq	SampleEf_BRP2_ST26_25ng_LIB4.R1.fastq.gz	SampleEf_BRP2_ST26_25ng_LIB4.R2.fastq.gz
BRP	SAMN17005115	SampleEfIS_BRP2_ST25_50ng_LIB1	paired	Illumina NovaSeq 6000		fastq	SampleEfIS_BRP2_ST25_50ng_LIB1.R1.fastq.gz	SampleEfIS_BRP2_ST25_50ng_LIB1.R2.fastq.gz
BRP	SAMN17005115	SampleEfIS_BRP2_ST25_50ng_LIB2	paired	Illumina NovaSeq 6000		fastq	SampleEfIS_BRP2_ST25_50ng_LIB2.R1.fastq.gz	SampleEfIS_BRP2_ST25_50ng_LIB2.R2.fastq.gz
BRP	SAMN17005115	SampleEfIS_BRP2_ST25_50ng_LIB3	paired	Illumina NovaSeq 6000		fastq	SampleEfIS_BRP2_ST25_50ng_LIB3.R1.fastq.gz	SampleEfIS_BRP2_ST25_50ng_LIB3.R2.fastq.gz
BRP	SAMN17005115	SampleEfIS_BRP2_ST25_50ng_LIB4	paired	Illumina NovaSeq 6000		fastq	SampleEfIS_BRP2_ST25_50ng_LIB4.R1.fastq.gz	SampleEfIS_BRP2_ST25_50ng_LIB4.R2.fastq.gz
BRP	SAMN17005115	SampleEfIS_BRP2_ST26_50ng_LIB1	paired	Illumina NovaSeq 6000		fastq	SampleEfIS_BRP2_ST26_50ng_LIB1.R1.fastq.gz	SampleEfIS_BRP2_ST26_50ng_LIB1.R2.fastq.gz
BRP	SAMN17005115	SampleEfIS_BRP2_ST26_50ng_LIB2	paired	Illumina NovaSeq 6000		fastq	SampleEfIS_BRP2_ST26_50ng_LIB2.R1.fastq.gz	SampleEfIS_BRP2_ST26_50ng_LIB2.R2.fastq.gz
BRP	SAMN17005115	SampleEfIS_BRP2_ST26_50ng_LIB3	paired	Illumina NovaSeq 6000		fastq	SampleEfIS_BRP2_ST26_50ng_LIB3.R1.fastq.gz	SampleEfIS_BRP2_ST26_50ng_LIB3.R2.fastq.gz
BRP	SAMN17005115	SampleEfIS_BRP2_ST26_50ng_LIB4	paired	Illumina NovaSeq 6000		fastq	SampleEfIS_BRP2_ST26_50ng_LIB4.R1.fastq.gz	SampleEfIS_BRP2_ST26_50ng_LIB4.R2.fastq.gz
BRP	SAMN17005116	SampleEp_BRP2_ST25_25ng_LIB1	paired	Illumina NovaSeq 6000		fastq	SampleEp_BRP2_ST25_25ng_LIB1.R1.fastq.gz	SampleEp_BRP2_ST25_25ng_LIB1.R2.fastq.gz
BRP	SAMN17005116	SampleEp_BRP2_ST25_25ng_LIB2	paired	Illumina NovaSeq 6000		fastq	SampleEp_BRP2_ST25_25ng_LIB2.R1.fastq.gz	SampleEp_BRP2_ST25_25ng_LIB2.R2.fastq.gz
BRP	SAMN17005116	SampleEp_BRP2_ST25_25ng_LIB3	paired	Illumina NovaSeq 6000		fastq	SampleEp_BRP2_ST25_25ng_LIB3.R1.fastq.gz	SampleEp_BRP2_ST25_25ng_LIB3.R2.fastq.gz
BRP	SAMN17005116	SampleEp_BRP2_ST25_25ng_LIB4	paired	Illumina NovaSeq 6000		fastq	SampleEp_BRP2_ST25_25ng_LIB4.R1.fastq.gz	SampleEp_BRP2_ST25_25ng_LIB4.R2.fastq.gz
BRP	SAMN17005116	SampleEp_BRP2_ST26_25ng_LIB1	paired	Illumina NovaSeq 6000		fastq	SampleEp_BRP2_ST26_25ng_LIB1.R1.fastq.gz	SampleEp_BRP2_ST26_25ng_LIB1.R2.fastq.gz
BRP	SAMN17005116	SampleEp_BRP2_ST26_25ng_LIB2	paired	Illumina NovaSeq 6000		fastq	SampleEp_BRP2_ST26_25ng_LIB2.R1.fastq.gz	SampleEp_BRP2_ST26_25ng_LIB2.R2.fastq.gz
BRP	SAMN17005116	SampleEp_BRP2_ST26_25ng_LIB3	paired	Illumina NovaSeq 6000		fastq	SampleEp_BRP2_ST26_25ng_LIB3.R1.fastq.gz	SampleEp_BRP2_ST26_25ng_LIB3.R2.fastq.gz
BRP	SAMN17005116	SampleEp_BRP2_ST26_25ng_LIB4	paired	Illumina NovaSeq 6000		fastq	SampleEp_BRP2_ST26_25ng_LIB4.R1.fastq.gz	SampleEp_BRP2_ST26_25ng_LIB4.R2.fastq.gz
IDT	SAMN17013275	SampleBfIS_IDT2_ST04_25ng_LIB1	paired	Illumina NovaSeq 6000		fastq	SampleBfIS_IDT2_ST04_25ng_LIB1.R1.fastq.gz	SampleBfIS_IDT2_ST04_25ng_LIB1.R2.fastq.gz
IDT	SAMN17013275	SampleBfIS_IDT2_ST04_25ng_LIB2	paired	Illumina NovaSeq 6000		fastq	SampleBfIS_IDT2_ST04_25ng_LIB2.R1.fastq.gz	SampleBfIS_IDT2_ST04_25ng_LIB2.R2.fastq.gz
IDT	SAMN17013275	SampleBfIS_IDT2_ST04_25ng_LIB3	paired	Illumina NovaSeq 6000		fastq	SampleBfIS_IDT2_ST04_25ng_LIB3.R1.fastq.gz	SampleBfIS_IDT2_ST04_25ng_LIB3.R2.fastq.gz
IDT	SAMN17013275	SampleBfIS_IDT2_ST04_25ng_LIB4	paired	Illumina NovaSeq 6000		fastq	SampleBfIS_IDT2_ST04_25ng_LIB4.R1.fastq.gz	SampleBfIS_IDT2_ST04_25ng_LIB4.R2.fastq.gz
IDT	SAMN17013275	SampleBfIS_IDT2_ST05_25ng_LIB1	paired	Illumina NovaSeq 6000		fastq	SampleBfIS_IDT2_ST05_25ng_LIB1.R1.fastq.gz	SampleBfIS_IDT2_ST05_25ng_LIB1.R2.fastq.gz
IDT	SAMN17013275	SampleBfIS_IDT2_ST05_25ng_LIB2	paired	Illumina NovaSeq 6000		fastq	SampleBfIS_IDT2_ST05_25ng_LIB2.R1.fastq.gz	SampleBfIS_IDT2_ST05_25ng_LIB2.R2.fastq.gz
IDT	SAMN17013275	SampleBfIS_IDT2_ST05_25ng_LIB3	paired	Illumina NovaSeq 6000		fastq	SampleBfIS_IDT2_ST05_25ng_LIB3.R1.fastq.gz	SampleBfIS_IDT2_ST05_25ng_LIB3.R2.fastq.gz
IDT	SAMN17013275	SampleBfIS_IDT2_ST05_25ng_LIB4	paired	Illumina NovaSeq 6000		fastq	SampleBfIS_IDT2_ST05_25ng_LIB4.R1.fastq.gz	SampleBfIS_IDT2_ST05_25ng_LIB4.R2.fastq.gz
IDT	SAMN17013275	SampleBfIS_IDT2_ST06_25ng_LIB1	paired	Illumina NovaSeq 6000		fastq	SampleBfIS_IDT2_ST06_25ng_LIB1.R1.fastq.gz	SampleBfIS_IDT2_ST06_25ng_LIB1.R2.fastq.gz
IDT	SAMN17013275	SampleBfIS_IDT2_ST06_25ng_LIB2	paired	Illumina NovaSeq 6000		fastq	SampleBfIS_IDT2_ST06_25ng_LIB2.R1.fastq.gz	SampleBfIS_IDT2_ST06_25ng_LIB2.R2.fastq.gz
IDT	SAMN17013275	SampleBfIS_IDT2_ST06_25ng_LIB3	paired	Illumina NovaSeq 6000		fastq	SampleBfIS_IDT2_ST06_25ng_LIB3.R1.fastq.gz	SampleBfIS_IDT2_ST06_25ng_LIB3.R2.fastq.gz
IDT	SAMN17013275	SampleBfIS_IDT2_ST06_25ng_LIB4	paired	Illumina NovaSeq 6000		fastq	SampleBfIS_IDT2_ST06_25ng_LIB4.R1.fastq.gz	SampleBfIS_IDT2_ST06_25ng_LIB4.R2.fastq.gz
IDT	SAMN17013276	SampleDfIS_IDT2_ST04_25ng_LIB1	paired	Illumina NovaSeq 6000		fastq	SampleDfIS_IDT2_ST04_25ng_LIB1.R1.fastq.gz	SampleDfIS_IDT2_ST04_25ng_LIB1.R2.fastq.gz
IDT	SAMN17013276	SampleDfIS_IDT2_ST04_25ng_LIB2	paired	Illumina NovaSeq 6000		fastq	SampleDfIS_IDT2_ST04_25ng_LIB2.R1.fastq.gz	SampleDfIS_IDT2_ST04_25ng_LIB2.R2.fastq.gz
IDT	SAMN17013276	SampleDfIS_IDT2_ST04_25ng_LIB3	paired	Illumina NovaSeq 6000		fastq	SampleDfIS_IDT2_ST04_25ng_LIB3.R1.fastq.gz	SampleDfIS_IDT2_ST04_25ng_LIB3.R2.fastq.gz
IDT	SAMN17013276	SampleDfIS_IDT2_ST04_25ng_LIB4	paired	Illumina NovaSeq 6000		fastq	SampleDfIS_IDT2_ST04_25ng_LIB4.R1.fastq.gz	SampleDfIS_IDT2_ST04_25ng_LIB4.R2.fastq.gz
IDT	SAMN17013276	SampleDfIS_IDT2_ST05_25ng_LIB1	paired	Illumina NovaSeq 6000		fastq	SampleDfIS_IDT2_ST05_25ng_LIB1.R1.fastq.gz	SampleDfIS_IDT2_ST05_25ng_LIB1.R2.fastq.gz
IDT	SAMN17013276	SampleDfIS_IDT2_ST05_25ng_LIB2	paired	Illumina NovaSeq 6000		fastq	SampleDfIS_IDT2_ST05_25ng_LIB2.R1.fastq.gz	SampleDfIS_IDT2_ST05_25ng_LIB2.R2.fastq.gz
IDT	SAMN17013276	SampleDfIS_IDT2_ST05_25ng_LIB3	paired	Illumina NovaSeq 6000		fastq	SampleDfIS_IDT2_ST05_25ng_LIB3.R1.fastq.gz	SampleDfIS_IDT2_ST05_25ng_LIB3.R2.fastq.gz
IDT	SAMN17013276	SampleDfIS_IDT2_ST05_25ng_LIB4	paired	Illumina NovaSeq 6000		fastq	SampleDfIS_IDT2_ST05_25ng_LIB4.R1.fastq.gz	SampleDfIS_IDT2_ST05_25ng_LIB4.R2.fastq.gz
IDT	SAMN17013276	SampleDfIS_IDT2_ST06_25ng_LIB1	paired	Illumina NovaSeq 6000		fastq	SampleDfIS_IDT2_ST06_25ng_LIB1.R1.fastq.gz	SampleDfIS_IDT2_ST06_25ng_LIB1.R2.fastq.gz
IDT	SAMN17013276	SampleDfIS_IDT2_ST06_25ng_LIB2	paired	Illumina NovaSeq 6000		fastq	SampleDfIS_IDT2_ST06_25ng_LIB2.R1.fastq.gz	SampleDfIS_IDT2_ST06_25ng_LIB2.R2.fastq.gz
IDT	SAMN17013276	SampleDfIS_IDT2_ST06_25ng_LIB3	paired	Illumina NovaSeq 6000		fastq	SampleDfIS_IDT2_ST06_25ng_LIB3.R1.fastq.gz	SampleDfIS_IDT2_ST06_25ng_LIB3.R2.fastq.gz
IDT	SAMN17013276	SampleDfIS_IDT2_ST06_25ng_LIB4	paired	Illumina NovaSeq 6000		fastq	SampleDfIS_IDT2_ST06_25ng_LIB4.R1.fastq.gz	SampleDfIS_IDT2_ST06_25ng_LIB4.R2.fastq.gz
IDT	SAMN17005115	SampleEfIS_IDT2_ST04_10ng_LIB1	paired	Illumina NovaSeq 6000		fastq	SampleEfIS_IDT2_ST04_10ng_LIB1.R1.fastq.gz	SampleEfIS_IDT2_ST04_10ng_LIB1.R2.fastq.gz
IDT	SAMN17005115	SampleEfIS_IDT2_ST04_10ng_LIB2	paired	Illumina NovaSeq 6000		fastq	SampleEfIS_IDT2_ST04_10ng_LIB2.R1.fastq.gz	SampleEfIS_IDT2_ST04_10ng_LIB2.R2.fastq.gz
IDT	SAMN17005115	SampleEfIS_IDT2_ST04_10ng_LIB3	paired	Illumina NovaSeq 6000		fastq	SampleEfIS_IDT2_ST04_10ng_LIB3.R1.fastq.gz	SampleEfIS_IDT2_ST04_10ng_LIB3.R2.fastq.gz
IDT	SAMN17005115	SampleEfIS_IDT2_ST04_10ng_LIB4	paired	Illumina NovaSeq 6000		fastq	SampleEfIS_IDT2_ST04_10ng_LIB4.R1.fastq.gz	SampleEfIS_IDT2_ST04_10ng_LIB4.R2.fastq.gz
IDT	SAMN17005115	SampleEfIS_IDT2_ST04_25ng_LIB1	paired	Illumina NovaSeq 6000		fastq	SampleEfIS_IDT2_ST04_25ng_LIB1.R1.fastq.gz	SampleEfIS_IDT2_ST04_25ng_LIB1.R2.fastq.gz
IDT	SAMN17005115	SampleEfIS_IDT2_ST04_25ng_LIB2	paired	Illumina NovaSeq 6000		fastq	SampleEfIS_IDT2_ST04_25ng_LIB2.R1.fastq.gz	SampleEfIS_IDT2_ST04_25ng_LIB2.R2.fastq.gz
IDT	SAMN17005115	SampleEfIS_IDT2_ST04_25ng_LIB3	paired	Illumina NovaSeq 6000		fastq	SampleEfIS_IDT2_ST04_25ng_LIB3.R1.fastq.gz	SampleEfIS_IDT2_ST04_25ng_LIB3.R2.fastq.gz
IDT	SAMN17005115	SampleEfIS_IDT2_ST04_25ng_LIB4	paired	Illumina NovaSeq 6000		fastq	SampleEfIS_IDT2_ST04_25ng_LIB4.R1.fastq.gz	SampleEfIS_IDT2_ST04_25ng_LIB4.R2.fastq.gz
IDT	SAMN17005115	SampleEfIS_IDT2_ST04_50ng_LIB1	paired	Illumina NovaSeq 6000		fastq	SampleEfIS_IDT2_ST04_50ng_LIB1.R1.fastq.gz	SampleEfIS_IDT2_ST04_50ng_LIB1.R2.fastq.gz
IDT	SAMN17005115	SampleEfIS_IDT2_ST04_50ng_LIB2	paired	Illumina NovaSeq 6000		fastq	SampleEfIS_IDT2_ST04_50ng_LIB2.R1.fastq.gz	SampleEfIS_IDT2_ST04_50ng_LIB2.R2.fastq.gz
IDT	SAMN17005115	SampleEfIS_IDT2_ST04_50ng_LIB3	paired	Illumina NovaSeq 6000		fastq	SampleEfIS_IDT2_ST04_50ng_LIB3.R1.fastq.gz	SampleEfIS_IDT2_ST04_50ng_LIB3.R2.fastq.gz
IDT	SAMN17005115	SampleEfIS_IDT2_ST04_50ng_LIB4	paired	Illumina NovaSeq 6000		fastq	SampleEfIS_IDT2_ST04_50ng_LIB4.R1.fastq.gz	SampleEfIS_IDT2_ST04_50ng_LIB4.R2.fastq.gz
IDT	SAMN17005115	SampleEfIS_IDT2_ST05_10ng_LIB1	paired	Illumina NovaSeq 6000		fastq	SampleEfIS_IDT2_ST05_10ng_LIB1.R1.fastq.gz	SampleEfIS_IDT2_ST05_10ng_LIB1.R2.fastq.gz
IDT	SAMN17005115	SampleEfIS_IDT2_ST05_10ng_LIB2	paired	Illumina NovaSeq 6000		fastq	SampleEfIS_IDT2_ST05_10ng_LIB2.R1.fastq.gz	SampleEfIS_IDT2_ST05_10ng_LIB2.R2.fastq.gz
IDT	SAMN17005115	SampleEfIS_IDT2_ST05_10ng_LIB3	paired	Illumina NovaSeq 6000		fastq	SampleEfIS_IDT2_ST05_10ng_LIB3.R1.fastq.gz	SampleEfIS_IDT2_ST05_10ng_LIB3.R2.fastq.gz
IDT	SAMN17005115	SampleEfIS_IDT2_ST05_10ng_LIB4	paired	Illumina NovaSeq 6000		fastq	SampleEfIS_IDT2_ST05_10ng_LIB4.R1.fastq.gz	SampleEfIS_IDT2_ST05_10ng_LIB4.R2.fastq.gz
IDT	SAMN17005115	SampleEfIS_IDT2_ST05_25ng_LIB1	paired	Illumina NovaSeq 6000		fastq	SampleEfIS_IDT2_ST05_25ng_LIB1.R1.fastq.gz	SampleEfIS_IDT2_ST05_25ng_LIB1.R2.fastq.gz
IDT	SAMN17005115	SampleEfIS_IDT2_ST05_25ng_LIB2	paired	Illumina NovaSeq 6000		fastq	SampleEfIS_IDT2_ST05_25ng_LIB2.R1.fastq.gz	SampleEfIS_IDT2_ST05_25ng_LIB2.R2.fastq.gz
IDT	SAMN17005115	SampleEfIS_IDT2_ST05_25ng_LIB3	paired	Illumina NovaSeq 6000		fastq	SampleEfIS_IDT2_ST05_25ng_LIB3.R1.fastq.gz	SampleEfIS_IDT2_ST05_25ng_LIB3.R2.fastq.gz
IDT	SAMN17005115	SampleEfIS_IDT2_ST05_25ng_LIB4	paired	Illumina NovaSeq 6000		fastq	SampleEfIS_IDT2_ST05_25ng_LIB4.R1.fastq.gz	SampleEfIS_IDT2_ST05_25ng_LIB4.R2.fastq.gz
IDT	SAMN17005115	SampleEfIS_IDT2_ST05_50ng_LIB1	paired	Illumina NovaSeq 6000		fastq	SampleEfIS_IDT2_ST05_50ng_LIB1.R1.fastq.gz	SampleEfIS_IDT2_ST05_50ng_LIB1.R2.fastq.gz
IDT	SAMN17005115	SampleEfIS_IDT2_ST05_50ng_LIB2	paired	Illumina NovaSeq 6000		fastq	SampleEfIS_IDT2_ST05_50ng_LIB2.R1.fastq.gz	SampleEfIS_IDT2_ST05_50ng_LIB2.R2.fastq.gz
IDT	SAMN17005115	SampleEfIS_IDT2_ST05_50ng_LIB3	paired	Illumina NovaSeq 6000		fastq	SampleEfIS_IDT2_ST05_50ng_LIB3.R1.fastq.gz	SampleEfIS_IDT2_ST05_50ng_LIB3.R2.fastq.gz
IDT	SAMN17005115	SampleEfIS_IDT2_ST05_50ng_LIB4	paired	Illumina NovaSeq 6000		fastq	SampleEfIS_IDT2_ST05_50ng_LIB4.R1.fastq.gz	SampleEfIS_IDT2_ST05_50ng_LIB4.R2.fastq.gz
IDT	SAMN17005115	SampleEfIS_IDT2_ST06_10ng_LIB1	paired	Illumina NovaSeq 6000		fastq	SampleEfIS_IDT2_ST06_10ng_LIB1.R1.fastq.gz	SampleEfIS_IDT2_ST06_10ng_LIB1.R2.fastq.gz
IDT	SAMN17005115	SampleEfIS_IDT2_ST06_10ng_LIB2	paired	Illumina NovaSeq 6000		fastq	SampleEfIS_IDT2_ST06_10ng_LIB2.R1.fastq.gz	SampleEfIS_IDT2_ST06_10ng_LIB2.R2.fastq.gz
IDT	SAMN17005115	SampleEfIS_IDT2_ST06_10ng_LIB3	paired	Illumina NovaSeq 6000		fastq	SampleEfIS_IDT2_ST06_10ng_LIB3.R1.fastq.gz	SampleEfIS_IDT2_ST06_10ng_LIB3.R2.fastq.gz
IDT	SAMN17005115	SampleEfIS_IDT2_ST06_10ng_LIB4	paired	Illumina NovaSeq 6000		fastq	SampleEfIS_IDT2_ST06_10ng_LIB4.R1.fastq.gz	SampleEfIS_IDT2_ST06_10ng_LIB4.R2.fastq.gz
IDT	SAMN17005115	SampleEfIS_IDT2_ST06_25ng_LIB1	paired	Illumina NovaSeq 6000		fastq	SampleEfIS_IDT2_ST06_25ng_LIB1.R1.fastq.gz	SampleEfIS_IDT2_ST06_25ng_LIB1.R2.fastq.gz
IDT	SAMN17005115	SampleEfIS_IDT2_ST06_25ng_LIB2	paired	Illumina NovaSeq 6000		fastq	SampleEfIS_IDT2_ST06_25ng_LIB2.R1.fastq.gz	SampleEfIS_IDT2_ST06_25ng_LIB2.R2.fastq.gz
IDT	SAMN17005115	SampleEfIS_IDT2_ST06_25ng_LIB3	paired	Illumina NovaSeq 6000		fastq	SampleEfIS_IDT2_ST06_25ng_LIB3.R1.fastq.gz	SampleEfIS_IDT2_ST06_25ng_LIB3.R2.fastq.gz
IDT	SAMN17005115	SampleEfIS_IDT2_ST06_25ng_LIB4	paired	Illumina NovaSeq 6000		fastq	SampleEfIS_IDT2_ST06_25ng_LIB4.R1.fastq.gz	SampleEfIS_IDT2_ST06_25ng_LIB4.R2.fastq.gz
IDT	SAMN17005115	SampleEfIS_IDT2_ST06_50ng_LIB1	paired	Illumina NovaSeq 6000		fastq	SampleEfIS_IDT2_ST06_50ng_LIB1.R1.fastq.gz	SampleEfIS_IDT2_ST06_50ng_LIB1.R2.fastq.gz
IDT	SAMN17005115	SampleEfIS_IDT2_ST06_50ng_LIB2	paired	Illumina NovaSeq 6000		fastq	SampleEfIS_IDT2_ST06_50ng_LIB2.R1.fastq.gz	SampleEfIS_IDT2_ST06_50ng_LIB2.R2.fastq.gz
IDT	SAMN17005115	SampleEfIS_IDT2_ST06_50ng_LIB3	paired	Illumina NovaSeq 6000		fastq	SampleEfIS_IDT2_ST06_50ng_LIB3.R1.fastq.gz	SampleEfIS_IDT2_ST06_50ng_LIB3.R2.fastq.gz
IDT	SAMN17005115	SampleEfIS_IDT2_ST06_50ng_LIB4	paired	Illumina NovaSeq 6000		fastq	SampleEfIS_IDT2_ST06_50ng_LIB4.R1.fastq.gz	SampleEfIS_IDT2_ST06_50ng_LIB4.R2.fastq.gz
IDT	SAMN17005116	SampleEp_IDT2_ST04_25ng_LIB1	paired	Illumina NovaSeq 6000		fastq	SampleEp_IDT2_ST04_25ng_LIB1.R1.fastq.gz	SampleEp_IDT2_ST04_25ng_LIB1.R2.fastq.gz
IDT	SAMN17005116	SampleEp_IDT2_ST04_25ng_LIB2	paired	Illumina NovaSeq 6000		fastq	SampleEp_IDT2_ST04_25ng_LIB2.R1.fastq.gz	SampleEp_IDT2_ST04_25ng_LIB2.R2.fastq.gz
IDT	SAMN17005116	SampleEp_IDT2_ST04_25ng_LIB3	paired	Illumina NovaSeq 6000		fastq	SampleEp_IDT2_ST04_25ng_LIB3.R1.fastq.gz	SampleEp_IDT2_ST04_25ng_LIB3.R2.fastq.gz
IDT	SAMN17005116	SampleEp_IDT2_ST04_25ng_LIB4	paired	Illumina NovaSeq 6000		fastq	SampleEp_IDT2_ST04_25ng_LIB4.R1.fastq.gz	SampleEp_IDT2_ST04_25ng_LIB4.R2.fastq.gz
IDT	SAMN17005116	SampleEp_IDT2_ST05_25ng_LIB1	paired	Illumina NovaSeq 6000		fastq	SampleEp_IDT2_ST05_25ng_LIB1.R1.fastq.gz	SampleEp_IDT2_ST05_25ng_LIB1.R2.fastq.gz
IDT	SAMN17005116	SampleEp_IDT2_ST05_25ng_LIB2	paired	Illumina NovaSeq 6000		fastq	SampleEp_IDT2_ST05_25ng_LIB2.R1.fastq.gz	SampleEp_IDT2_ST05_25ng_LIB2.R2.fastq.gz
IDT	SAMN17005116	SampleEp_IDT2_ST05_25ng_LIB3	paired	Illumina NovaSeq 6000		fastq	SampleEp_IDT2_ST05_25ng_LIB3.R1.fastq.gz	SampleEp_IDT2_ST05_25ng_LIB3.R2.fastq.gz
IDT	SAMN17005116	SampleEp_IDT2_ST05_25ng_LIB4	paired	Illumina NovaSeq 6000		fastq	SampleEp_IDT2_ST05_25ng_LIB4.R1.fastq.gz	SampleEp_IDT2_ST05_25ng_LIB4.R2.fastq.gz
IDT	SAMN17005116	SampleEp_IDT2_ST06_25ng_LIB1	paired	Illumina NovaSeq 6000		fastq	SampleEp_IDT2_ST06_25ng_LIB1.R1.fastq.gz	SampleEp_IDT2_ST06_25ng_LIB1.R2.fastq.gz
IDT	SAMN17005116	SampleEp_IDT2_ST06_25ng_LIB2	paired	Illumina NovaSeq 6000		fastq	SampleEp_IDT2_ST06_25ng_LIB2.R1.fastq.gz	SampleEp_IDT2_ST06_25ng_LIB2.R2.fastq.gz
IDT	SAMN17005116	SampleEp_IDT2_ST06_25ng_LIB3	paired	Illumina NovaSeq 6000		fastq	SampleEp_IDT2_ST06_25ng_LIB3.R1.fastq.gz	SampleEp_IDT2_ST06_25ng_LIB3.R2.fastq.gz
IDT	SAMN17005116	SampleEp_IDT2_ST06_25ng_LIB4	paired	Illumina NovaSeq 6000		fastq	SampleEp_IDT2_ST06_25ng_LIB4.R1.fastq.gz	SampleEp_IDT2_ST06_25ng_LIB4.R2.fastq.gz
ILM	SAMN16786373	SampleBf_ILM2_ST10_25ng_LIB1	paired	Illumina NovaSeq 6000		fastq	SampleBf_ILM2_ST10_25ng_LIB1.R1.fastq.gz	SampleBf_ILM2_ST10_25ng_LIB1.R2.fastq.gz
ILM	SAMN16786373	SampleBf_ILM2_ST10_25ng_LIB2	paired	Illumina NovaSeq 6000		fastq	SampleBf_ILM2_ST10_25ng_LIB2.R1.fastq.gz	SampleBf_ILM2_ST10_25ng_LIB2.R2.fastq.gz
ILM	SAMN16786373	SampleBf_ILM2_ST10_25ng_LIB3	paired	Illumina NovaSeq 6000		fastq	SampleBf_ILM2_ST10_25ng_LIB3.R1.fastq.gz	SampleBf_ILM2_ST10_25ng_LIB3.R2.fastq.gz
ILM	SAMN16786373	SampleBf_ILM2_ST10_25ng_LIB4	paired	Illumina NovaSeq 6000		fastq	SampleBf_ILM2_ST10_25ng_LIB4.R1.fastq.gz	SampleBf_ILM2_ST10_25ng_LIB4.R2.fastq.gz
ILM	SAMN16786373	SampleBf_ILM2_ST23_25ng_LIB1	paired	Illumina NovaSeq 6000		fastq	SampleBf_ILM2_ST23_25ng_LIB1.R1.fastq.gz	SampleBf_ILM2_ST23_25ng_LIB1.R2.fastq.gz
ILM	SAMN16786373	SampleBf_ILM2_ST23_25ng_LIB2	paired	Illumina NovaSeq 6000		fastq	SampleBf_ILM2_ST23_25ng_LIB2.R1.fastq.gz	SampleBf_ILM2_ST23_25ng_LIB2.R2.fastq.gz
ILM	SAMN16786373	SampleBf_ILM2_ST23_25ng_LIB3	paired	Illumina NovaSeq 6000		fastq	SampleBf_ILM2_ST23_25ng_LIB3.R1.fastq.gz	SampleBf_ILM2_ST23_25ng_LIB3.R2.fastq.gz
ILM	SAMN16786373	SampleBf_ILM2_ST23_25ng_LIB4	paired	Illumina NovaSeq 6000		fastq	SampleBf_ILM2_ST23_25ng_LIB4.R1.fastq.gz	SampleBf_ILM2_ST23_25ng_LIB4.R2.fastq.gz
ILM	SAMN16786373	SampleBf_ILM2_ST29_25ng_LIB1	paired	Illumina NovaSeq 6000		fastq	SampleBf_ILM2_ST29_25ng_LIB1.R1.fastq.gz	SampleBf_ILM2_ST29_25ng_LIB1.R2.fastq.gz
ILM	SAMN16786373	SampleBf_ILM2_ST29_25ng_LIB2	paired	Illumina NovaSeq 6000		fastq	SampleBf_ILM2_ST29_25ng_LIB2.R1.fastq.gz	SampleBf_ILM2_ST29_25ng_LIB2.R2.fastq.gz
ILM	SAMN16786373	SampleBf_ILM2_ST29_25ng_LIB3	paired	Illumina NovaSeq 6000		fastq	SampleBf_ILM2_ST29_25ng_LIB3.R1.fastq.gz	SampleBf_ILM2_ST29_25ng_LIB3.R2.fastq.gz
ILM	SAMN16786373	SampleBf_ILM2_ST29_25ng_LIB4	paired	Illumina NovaSeq 6000		fastq	SampleBf_ILM2_ST29_25ng_LIB4.R1.fastq.gz	SampleBf_ILM2_ST29_25ng_LIB4.R2.fastq.gz
ILM	SAMN16786374	SampleDf_ILM2_ST10_25ng_LIB1	paired	Illumina NovaSeq 6000		fastq	SampleDf_ILM2_ST10_25ng_LIB1.R1.fastq.gz	SampleDf_ILM2_ST10_25ng_LIB1.R2.fastq.gz
ILM	SAMN16786374	SampleDf_ILM2_ST10_25ng_LIB2	paired	Illumina NovaSeq 6000		fastq	SampleDf_ILM2_ST10_25ng_LIB2.R1.fastq.gz	SampleDf_ILM2_ST10_25ng_LIB2.R2.fastq.gz
ILM	SAMN16786374	SampleDf_ILM2_ST10_25ng_LIB3	paired	Illumina NovaSeq 6000		fastq	SampleDf_ILM2_ST10_25ng_LIB3.R1.fastq.gz	SampleDf_ILM2_ST10_25ng_LIB3.R2.fastq.gz
ILM	SAMN16786374	SampleDf_ILM2_ST10_25ng_LIB4	paired	Illumina NovaSeq 6000		fastq	SampleDf_ILM2_ST10_25ng_LIB4.R1.fastq.gz	SampleDf_ILM2_ST10_25ng_LIB4.R2.fastq.gz
ILM	SAMN16786374	SampleDf_ILM2_ST23_25ng_LIB1	paired	Illumina NovaSeq 6000		fastq	SampleDf_ILM2_ST23_25ng_LIB1.R1.fastq.gz	SampleDf_ILM2_ST23_25ng_LIB1.R2.fastq.gz
ILM	SAMN16786374	SampleDf_ILM2_ST23_25ng_LIB2	paired	Illumina NovaSeq 6000		fastq	SampleDf_ILM2_ST23_25ng_LIB2.R1.fastq.gz	SampleDf_ILM2_ST23_25ng_LIB2.R2.fastq.gz
ILM	SAMN16786374	SampleDf_ILM2_ST23_25ng_LIB3	paired	Illumina NovaSeq 6000		fastq	SampleDf_ILM2_ST23_25ng_LIB3.R1.fastq.gz	SampleDf_ILM2_ST23_25ng_LIB3.R2.fastq.gz
ILM	SAMN16786374	SampleDf_ILM2_ST23_25ng_LIB4	paired	Illumina NovaSeq 6000		fastq	SampleDf_ILM2_ST23_25ng_LIB4.R1.fastq.gz	SampleDf_ILM2_ST23_25ng_LIB4.R2.fastq.gz
ILM	SAMN16786374	SampleDf_ILM2_ST29_25ng_LIB1	paired	Illumina NovaSeq 6000		fastq	SampleDf_ILM2_ST29_25ng_LIB1.R1.fastq.gz	SampleDf_ILM2_ST29_25ng_LIB1.R2.fastq.gz
ILM	SAMN16786374	SampleDf_ILM2_ST29_25ng_LIB2	paired	Illumina NovaSeq 6000		fastq	SampleDf_ILM2_ST29_25ng_LIB2.R1.fastq.gz	SampleDf_ILM2_ST29_25ng_LIB2.R2.fastq.gz
ILM	SAMN16786374	SampleDf_ILM2_ST29_25ng_LIB3	paired	Illumina NovaSeq 6000		fastq	SampleDf_ILM2_ST29_25ng_LIB3.R1.fastq.gz	SampleDf_ILM2_ST29_25ng_LIB3.R2.fastq.gz
ILM	SAMN16786374	SampleDf_ILM2_ST29_25ng_LIB4	paired	Illumina NovaSeq 6000		fastq	SampleDf_ILM2_ST29_25ng_LIB4.R1.fastq.gz	SampleDf_ILM2_ST29_25ng_LIB4.R2.fastq.gz
ILM	SAMN16786375	SampleEf_ILM2_ST10_10ng_LIB1	paired	Illumina NovaSeq 6000		fastq	SampleEf_ILM2_ST10_10ng_LIB1.R1.fastq.gz	SampleEf_ILM2_ST10_10ng_LIB1.R2.fastq.gz
ILM	SAMN16786375	SampleEf_ILM2_ST10_10ng_LIB2	paired	Illumina NovaSeq 6000		fastq	SampleEf_ILM2_ST10_10ng_LIB2.R1.fastq.gz	SampleEf_ILM2_ST10_10ng_LIB2.R2.fastq.gz
ILM	SAMN16786375	SampleEf_ILM2_ST10_10ng_LIB3	paired	Illumina NovaSeq 6000		fastq	SampleEf_ILM2_ST10_10ng_LIB3.R1.fastq.gz	SampleEf_ILM2_ST10_10ng_LIB3.R2.fastq.gz
ILM	SAMN16786375	SampleEf_ILM2_ST10_10ng_LIB4	paired	Illumina NovaSeq 6000		fastq	SampleEf_ILM2_ST10_10ng_LIB4.R1.fastq.gz	SampleEf_ILM2_ST10_10ng_LIB4.R2.fastq.gz
ILM	SAMN16786375	SampleEf_ILM2_ST10_25ng_LIB1	paired	Illumina NovaSeq 6000		fastq	SampleEf_ILM2_ST10_25ng_LIB1.R1.fastq.gz	SampleEf_ILM2_ST10_25ng_LIB1.R2.fastq.gz
ILM	SAMN16786375	SampleEf_ILM2_ST10_25ng_LIB2	paired	Illumina NovaSeq 6000		fastq	SampleEf_ILM2_ST10_25ng_LIB2.R1.fastq.gz	SampleEf_ILM2_ST10_25ng_LIB2.R2.fastq.gz
ILM	SAMN16786375	SampleEf_ILM2_ST10_25ng_LIB3	paired	Illumina NovaSeq 6000		fastq	SampleEf_ILM2_ST10_25ng_LIB3.R1.fastq.gz	SampleEf_ILM2_ST10_25ng_LIB3.R2.fastq.gz
ILM	SAMN16786375	SampleEf_ILM2_ST10_25ng_LIB4	paired	Illumina NovaSeq 6000		fastq	SampleEf_ILM2_ST10_25ng_LIB4.R1.fastq.gz	SampleEf_ILM2_ST10_25ng_LIB4.R2.fastq.gz
ILM	SAMN16786375	SampleEf_ILM2_ST23_10ng_LIB1	paired	Illumina NovaSeq 6000		fastq	SampleEf_ILM2_ST23_10ng_LIB1.R1.fastq.gz	SampleEf_ILM2_ST23_10ng_LIB1.R2.fastq.gz
ILM	SAMN16786375	SampleEf_ILM2_ST23_10ng_LIB2	paired	Illumina NovaSeq 6000		fastq	SampleEf_ILM2_ST23_10ng_LIB2.R1.fastq.gz	SampleEf_ILM2_ST23_10ng_LIB2.R2.fastq.gz
ILM	SAMN16786375	SampleEf_ILM2_ST23_10ng_LIB3	paired	Illumina NovaSeq 6000		fastq	SampleEf_ILM2_ST23_10ng_LIB3.R1.fastq.gz	SampleEf_ILM2_ST23_10ng_LIB3.R2.fastq.gz
ILM	SAMN16786375	SampleEf_ILM2_ST23_10ng_LIB4	paired	Illumina NovaSeq 6000		fastq	SampleEf_ILM2_ST23_10ng_LIB4.R1.fastq.gz	SampleEf_ILM2_ST23_10ng_LIB4.R2.fastq.gz
ILM	SAMN16786375	SampleEf_ILM2_ST23_25ng_LIB1	paired	Illumina NovaSeq 6000		fastq	SampleEf_ILM2_ST23_25ng_LIB1.R1.fastq.gz	SampleEf_ILM2_ST23_25ng_LIB1.R2.fastq.gz
ILM	SAMN16786375	SampleEf_ILM2_ST23_25ng_LIB2	paired	Illumina NovaSeq 6000		fastq	SampleEf_ILM2_ST23_25ng_LIB2.R1.fastq.gz	SampleEf_ILM2_ST23_25ng_LIB2.R2.fastq.gz
ILM	SAMN16786375	SampleEf_ILM2_ST23_25ng_LIB3	paired	Illumina NovaSeq 6000		fastq	SampleEf_ILM2_ST23_25ng_LIB3.R1.fastq.gz	SampleEf_ILM2_ST23_25ng_LIB3.R2.fastq.gz
ILM	SAMN16786375	SampleEf_ILM2_ST23_25ng_LIB4	paired	Illumina NovaSeq 6000		fastq	SampleEf_ILM2_ST23_25ng_LIB4.R1.fastq.gz	SampleEf_ILM2_ST23_25ng_LIB4.R2.fastq.gz
ILM	SAMN16786375	SampleEf_ILM2_ST29_10ng_LIB1	paired	Illumina NovaSeq 6000		fastq	SampleEf_ILM2_ST29_10ng_LIB1.R1.fastq.gz	SampleEf_ILM2_ST29_10ng_LIB1.R2.fastq.gz
ILM	SAMN16786375	SampleEf_ILM2_ST29_10ng_LIB2	paired	Illumina NovaSeq 6000		fastq	SampleEf_ILM2_ST29_10ng_LIB2.R1.fastq.gz	SampleEf_ILM2_ST29_10ng_LIB2.R2.fastq.gz
ILM	SAMN16786375	SampleEf_ILM2_ST29_10ng_LIB3	paired	Illumina NovaSeq 6000		fastq	SampleEf_ILM2_ST29_10ng_LIB3.R1.fastq.gz	SampleEf_ILM2_ST29_10ng_LIB3.R2.fastq.gz
ILM	SAMN16786375	SampleEf_ILM2_ST29_10ng_LIB4	paired	Illumina NovaSeq 6000		fastq	SampleEf_ILM2_ST29_10ng_LIB4.R1.fastq.gz	SampleEf_ILM2_ST29_10ng_LIB4.R2.fastq.gz
ILM	SAMN16786375	SampleEf_ILM2_ST29_25ng_LIB1	paired	Illumina NovaSeq 6000		fastq	SampleEf_ILM2_ST29_25ng_LIB1.R1.fastq.gz	SampleEf_ILM2_ST29_25ng_LIB1.R2.fastq.gz
ILM	SAMN16786375	SampleEf_ILM2_ST29_25ng_LIB2	paired	Illumina NovaSeq 6000		fastq	SampleEf_ILM2_ST29_25ng_LIB2.R1.fastq.gz	SampleEf_ILM2_ST29_25ng_LIB2.R2.fastq.gz
ILM	SAMN16786375	SampleEf_ILM2_ST29_25ng_LIB3	paired	Illumina NovaSeq 6000		fastq	SampleEf_ILM2_ST29_25ng_LIB3.R1.fastq.gz	SampleEf_ILM2_ST29_25ng_LIB3.R2.fastq.gz
ILM	SAMN16786375	SampleEf_ILM2_ST29_25ng_LIB4	paired	Illumina NovaSeq 6000		fastq	SampleEf_ILM2_ST29_25ng_LIB4.R1.fastq.gz	SampleEf_ILM2_ST29_25ng_LIB4.R2.fastq.gz
ILM	SAMN17005115	SampleEfIS_ILM2_ST10_50ng_LIB1	paired	Illumina NovaSeq 6000		fastq	SampleEfIS_ILM2_ST10_50ng_LIB1.R1.fastq.gz	SampleEfIS_ILM2_ST10_50ng_LIB1.R2.fastq.gz
ILM	SAMN17005115	SampleEfIS_ILM2_ST10_50ng_LIB2	paired	Illumina NovaSeq 6000		fastq	SampleEfIS_ILM2_ST10_50ng_LIB2.R1.fastq.gz	SampleEfIS_ILM2_ST10_50ng_LIB2.R2.fastq.gz
ILM	SAMN17005115	SampleEfIS_ILM2_ST10_50ng_LIB3	paired	Illumina NovaSeq 6000		fastq	SampleEfIS_ILM2_ST10_50ng_LIB3.R1.fastq.gz	SampleEfIS_ILM2_ST10_50ng_LIB3.R2.fastq.gz
ILM	SAMN17005115	SampleEfIS_ILM2_ST10_50ng_LIB4	paired	Illumina NovaSeq 6000		fastq	SampleEfIS_ILM2_ST10_50ng_LIB4.R1.fastq.gz	SampleEfIS_ILM2_ST10_50ng_LIB4.R2.fastq.gz
ILM	SAMN17005115	SampleEfIS_ILM2_ST23_50ng_LIB1	paired	Illumina NovaSeq 6000		fastq	SampleEfIS_ILM2_ST23_50ng_LIB1.R1.fastq.gz	SampleEfIS_ILM2_ST23_50ng_LIB1.R2.fastq.gz
ILM	SAMN17005115	SampleEfIS_ILM2_ST23_50ng_LIB2	paired	Illumina NovaSeq 6000		fastq	SampleEfIS_ILM2_ST23_50ng_LIB2.R1.fastq.gz	SampleEfIS_ILM2_ST23_50ng_LIB2.R2.fastq.gz
ILM	SAMN17005115	SampleEfIS_ILM2_ST23_50ng_LIB3	paired	Illumina NovaSeq 6000		fastq	SampleEfIS_ILM2_ST23_50ng_LIB3.R1.fastq.gz	SampleEfIS_ILM2_ST23_50ng_LIB3.R2.fastq.gz
ILM	SAMN17005115	SampleEfIS_ILM2_ST23_50ng_LIB4	paired	Illumina NovaSeq 6000		fastq	SampleEfIS_ILM2_ST23_50ng_LIB4.R1.fastq.gz	SampleEfIS_ILM2_ST23_50ng_LIB4.R2.fastq.gz
ILM	SAMN17005115	SampleEfIS_ILM2_ST29_50ng_LIB1	paired	Illumina NovaSeq 6000		fastq	SampleEfIS_ILM2_ST29_50ng_LIB1.R1.fastq.gz	SampleEfIS_ILM2_ST29_50ng_LIB1.R2.fastq.gz
ILM	SAMN17005115	SampleEfIS_ILM2_ST29_50ng_LIB2	paired	Illumina NovaSeq 6000		fastq	SampleEfIS_ILM2_ST29_50ng_LIB2.R1.fastq.gz	SampleEfIS_ILM2_ST29_50ng_LIB2.R2.fastq.gz
ILM	SAMN17005115	SampleEfIS_ILM2_ST29_50ng_LIB3	paired	Illumina NovaSeq 6000		fastq	SampleEfIS_ILM2_ST29_50ng_LIB3.R1.fastq.gz	SampleEfIS_ILM2_ST29_50ng_LIB3.R2.fastq.gz
ILM	SAMN17005115	SampleEfIS_ILM2_ST29_50ng_LIB4	paired	Illumina NovaSeq 6000		fastq	SampleEfIS_ILM2_ST29_50ng_LIB4.R1.fastq.gz	SampleEfIS_ILM2_ST29_50ng_LIB4.R2.fastq.gz
ILM	SAMN17005116	SampleEp_ILM2_ST10_25ng_LIB1	paired	Illumina NovaSeq 6000		fastq	SampleEp_ILM2_ST10_25ng_LIB1.R1.fastq.gz	SampleEp_ILM2_ST10_25ng_LIB1.R2.fastq.gz
ILM	SAMN17005116	SampleEp_ILM2_ST10_25ng_LIB2	paired	Illumina NovaSeq 6000		fastq	SampleEp_ILM2_ST10_25ng_LIB2.R1.fastq.gz	SampleEp_ILM2_ST10_25ng_LIB2.R2.fastq.gz
ILM	SAMN17005116	SampleEp_ILM2_ST10_25ng_LIB3	paired	Illumina NovaSeq 6000		fastq	SampleEp_ILM2_ST10_25ng_LIB3.R1.fastq.gz	SampleEp_ILM2_ST10_25ng_LIB3.R2.fastq.gz
ILM	SAMN17005116	SampleEp_ILM2_ST10_25ng_LIB4	paired	Illumina NovaSeq 6000		fastq	SampleEp_ILM2_ST10_25ng_LIB4.R1.fastq.gz	SampleEp_ILM2_ST10_25ng_LIB4.R2.fastq.gz
ILM	SAMN17005116	SampleEp_ILM2_ST23_25ng_LIB1	paired	Illumina NovaSeq 6000		fastq	SampleEp_ILM2_ST23_25ng_LIB1.R1.fastq.gz	SampleEp_ILM2_ST23_25ng_LIB1.R2.fastq.gz
ILM	SAMN17005116	SampleEp_ILM2_ST23_25ng_LIB2	paired	Illumina NovaSeq 6000		fastq	SampleEp_ILM2_ST23_25ng_LIB2.R1.fastq.gz	SampleEp_ILM2_ST23_25ng_LIB2.R2.fastq.gz
ILM	SAMN17005116	SampleEp_ILM2_ST23_25ng_LIB3	paired	Illumina NovaSeq 6000		fastq	SampleEp_ILM2_ST23_25ng_LIB3.R1.fastq.gz	SampleEp_ILM2_ST23_25ng_LIB3.R2.fastq.gz
ILM	SAMN17005116	SampleEp_ILM2_ST23_25ng_LIB4	paired	Illumina NovaSeq 6000		fastq	SampleEp_ILM2_ST23_25ng_LIB4.R1.fastq.gz	SampleEp_ILM2_ST23_25ng_LIB4.R2.fastq.gz
ILM	SAMN17005116	SampleEp_ILM2_ST29_25ng_LIB1	paired	Illumina NovaSeq 6000		fastq	SampleEp_ILM2_ST29_25ng_LIB1.R1.fastq.gz	SampleEp_ILM2_ST29_25ng_LIB1.R2.fastq.gz
ILM	SAMN17005116	SampleEp_ILM2_ST29_25ng_LIB2	paired	Illumina NovaSeq 6000		fastq	SampleEp_ILM2_ST29_25ng_LIB2.R1.fastq.gz	SampleEp_ILM2_ST29_25ng_LIB2.R2.fastq.gz
ILM	SAMN17005116	SampleEp_ILM2_ST29_25ng_LIB3	paired	Illumina NovaSeq 6000		fastq	SampleEp_ILM2_ST29_25ng_LIB3.R1.fastq.gz	SampleEp_ILM2_ST29_25ng_LIB3.R2.fastq.gz
ILM	SAMN17005116	SampleEp_ILM2_ST29_25ng_LIB4	paired	Illumina NovaSeq 6000		fastq	SampleEp_ILM2_ST29_25ng_LIB4.R1.fastq.gz	SampleEp_ILM2_ST29_25ng_LIB4.R2.fastq.gz
ROC	SAMN16786373	SampleBf_ROC2_ST10_25ng_LIB1	paired	NextSeq 500		bam	SampleBf_ROC2_ST10_25ng_LIB1.barcode_deduplicated.bam	SampleBf_ROC2_ST10_25ng_LIB1.position_deduplicated.bam
ROC	SAMN16786373	SampleBf_ROC2_ST10_25ng_LIB2	paired	NextSeq 500		bam	SampleBf_ROC2_ST10_25ng_LIB2.barcode_deduplicated.bam	SampleBf_ROC2_ST10_25ng_LIB2.position_deduplicated.bam
ROC	SAMN16786373	SampleBf_ROC2_ST10_25ng_LIB3	paired	NextSeq 500		bam	SampleBf_ROC2_ST10_25ng_LIB3.barcode_deduplicated.bam	SampleBf_ROC2_ST10_25ng_LIB3.position_deduplicated.bam
ROC	SAMN16786373	SampleBf_ROC2_ST10_25ng_LIB4	paired	NextSeq 500		bam	SampleBf_ROC2_ST10_25ng_LIB4.barcode_deduplicated.bam	SampleBf_ROC2_ST10_25ng_LIB4.position_deduplicated.bam
ROC	SAMN16786373	SampleBf_ROC2_ST20_25ng_LIB1	paired	NextSeq 500		bam	SampleBf_ROC2_ST20_25ng_LIB1.barcode_deduplicated.bam	SampleBf_ROC2_ST20_25ng_LIB1.position_deduplicated.bam
ROC	SAMN16786373	SampleBf_ROC2_ST20_25ng_LIB2	paired	NextSeq 500		bam	SampleBf_ROC2_ST20_25ng_LIB2.barcode_deduplicated.bam	SampleBf_ROC2_ST20_25ng_LIB2.position_deduplicated.bam
ROC	SAMN16786373	SampleBf_ROC2_ST20_25ng_LIB3	paired	NextSeq 500		bam	SampleBf_ROC2_ST20_25ng_LIB3.barcode_deduplicated.bam	SampleBf_ROC2_ST20_25ng_LIB3.position_deduplicated.bam
ROC	SAMN16786373	SampleBf_ROC2_ST20_25ng_LIB4	paired	NextSeq 500		bam	SampleBf_ROC2_ST20_25ng_LIB4.barcode_deduplicated.bam	SampleBf_ROC2_ST20_25ng_LIB4.position_deduplicated.bam
ROC	SAMN16786373	SampleBf_ROC2_ST21_25ng_LIB1	paired	NextSeq 500		bam	SampleBf_ROC2_ST21_25ng_LIB1.barcode_deduplicated.bam	SampleBf_ROC2_ST21_25ng_LIB1.position_deduplicated.bam
ROC	SAMN16786373	SampleBf_ROC2_ST21_25ng_LIB2	paired	NextSeq 500		bam	SampleBf_ROC2_ST21_25ng_LIB2.barcode_deduplicated.bam	SampleBf_ROC2_ST21_25ng_LIB2.position_deduplicated.bam
ROC	SAMN16786373	SampleBf_ROC2_ST21_25ng_LIB3	paired	NextSeq 500		bam	SampleBf_ROC2_ST21_25ng_LIB3.barcode_deduplicated.bam	SampleBf_ROC2_ST21_25ng_LIB3.position_deduplicated.bam
ROC	SAMN16786373	SampleBf_ROC2_ST21_25ng_LIB4	paired	NextSeq 500		bam	SampleBf_ROC2_ST21_25ng_LIB4.barcode_deduplicated.bam	SampleBf_ROC2_ST21_25ng_LIB4.position_deduplicated.bam
ROC	SAMN16786374	SampleDf_ROC2_ST10_25ng_LIB1	paired	NextSeq 500		bam	SampleDf_ROC2_ST10_25ng_LIB1.barcode_deduplicated.bam	SampleDf_ROC2_ST10_25ng_LIB1.position_deduplicated.bam
ROC	SAMN16786374	SampleDf_ROC2_ST10_25ng_LIB2	paired	NextSeq 500		bam	SampleDf_ROC2_ST10_25ng_LIB2.barcode_deduplicated.bam	SampleDf_ROC2_ST10_25ng_LIB2.position_deduplicated.bam
ROC	SAMN16786374	SampleDf_ROC2_ST10_25ng_LIB3	paired	NextSeq 500		bam	SampleDf_ROC2_ST10_25ng_LIB3.barcode_deduplicated.bam	SampleDf_ROC2_ST10_25ng_LIB3.position_deduplicated.bam
ROC	SAMN16786374	SampleDf_ROC2_ST10_25ng_LIB4	paired	NextSeq 500		bam	SampleDf_ROC2_ST10_25ng_LIB4.barcode_deduplicated.bam	SampleDf_ROC2_ST10_25ng_LIB4.position_deduplicated.bam
ROC	SAMN16786374	SampleDf_ROC2_ST20_25ng_LIB1	paired	NextSeq 500		bam	SampleDf_ROC2_ST20_25ng_LIB1.barcode_deduplicated.bam	SampleDf_ROC2_ST20_25ng_LIB1.position_deduplicated.bam
ROC	SAMN16786374	SampleDf_ROC2_ST20_25ng_LIB2	paired	NextSeq 500		bam	SampleDf_ROC2_ST20_25ng_LIB2.barcode_deduplicated.bam	SampleDf_ROC2_ST20_25ng_LIB2.position_deduplicated.bam
ROC	SAMN16786374	SampleDf_ROC2_ST20_25ng_LIB3	paired	NextSeq 500		bam	SampleDf_ROC2_ST20_25ng_LIB3.barcode_deduplicated.bam	SampleDf_ROC2_ST20_25ng_LIB3.position_deduplicated.bam
ROC	SAMN16786374	SampleDf_ROC2_ST20_25ng_LIB4	paired	NextSeq 500		bam	SampleDf_ROC2_ST20_25ng_LIB4.barcode_deduplicated.bam	SampleDf_ROC2_ST20_25ng_LIB4.position_deduplicated.bam
ROC	SAMN16786374	SampleDf_ROC2_ST21_25ng_LIB1	paired	NextSeq 500		bam	SampleDf_ROC2_ST21_25ng_LIB1.barcode_deduplicated.bam	SampleDf_ROC2_ST21_25ng_LIB1.position_deduplicated.bam
ROC	SAMN16786374	SampleDf_ROC2_ST21_25ng_LIB2	paired	NextSeq 500		bam	SampleDf_ROC2_ST21_25ng_LIB2.barcode_deduplicated.bam	SampleDf_ROC2_ST21_25ng_LIB2.position_deduplicated.bam
ROC	SAMN16786374	SampleDf_ROC2_ST21_25ng_LIB3	paired	NextSeq 500		bam	SampleDf_ROC2_ST21_25ng_LIB3.barcode_deduplicated.bam	SampleDf_ROC2_ST21_25ng_LIB3.position_deduplicated.bam
ROC	SAMN16786374	SampleDf_ROC2_ST21_25ng_LIB4	paired	NextSeq 500		bam	SampleDf_ROC2_ST21_25ng_LIB4.barcode_deduplicated.bam	SampleDf_ROC2_ST21_25ng_LIB4.position_deduplicated.bam
ROC	SAMN16786375	SampleEf_ROC2_ST10_10ng_LIB1	paired	NextSeq 500		bam	SampleEf_ROC2_ST10_10ng_LIB1.barcode_deduplicated.bam	SampleEf_ROC2_ST10_10ng_LIB1.position_deduplicated.bam
ROC	SAMN16786375	SampleEf_ROC2_ST10_10ng_LIB2	paired	NextSeq 500		bam	SampleEf_ROC2_ST10_10ng_LIB2.barcode_deduplicated.bam	SampleEf_ROC2_ST10_10ng_LIB2.position_deduplicated.bam
ROC	SAMN16786375	SampleEf_ROC2_ST10_10ng_LIB3	paired	NextSeq 500		bam	SampleEf_ROC2_ST10_10ng_LIB3.barcode_deduplicated.bam	SampleEf_ROC2_ST10_10ng_LIB3.position_deduplicated.bam
ROC	SAMN16786375	SampleEf_ROC2_ST10_10ng_LIB4	paired	NextSeq 500		bam	SampleEf_ROC2_ST10_10ng_LIB4.barcode_deduplicated.bam	SampleEf_ROC2_ST10_10ng_LIB4.position_deduplicated.bam
ROC	SAMN16786375	SampleEf_ROC2_ST10_25ng_LIB1	paired	NextSeq 500		bam	SampleEf_ROC2_ST10_25ng_LIB1.barcode_deduplicated.bam	SampleEf_ROC2_ST10_25ng_LIB1.position_deduplicated.bam
ROC	SAMN16786375	SampleEf_ROC2_ST10_25ng_LIB2	paired	NextSeq 500		bam	SampleEf_ROC2_ST10_25ng_LIB2.barcode_deduplicated.bam	SampleEf_ROC2_ST10_25ng_LIB2.position_deduplicated.bam
ROC	SAMN16786375	SampleEf_ROC2_ST10_25ng_LIB3	paired	NextSeq 500		bam	SampleEf_ROC2_ST10_25ng_LIB3.barcode_deduplicated.bam	SampleEf_ROC2_ST10_25ng_LIB3.position_deduplicated.bam
ROC	SAMN16786375	SampleEf_ROC2_ST10_25ng_LIB4	paired	NextSeq 500		bam	SampleEf_ROC2_ST10_25ng_LIB4.barcode_deduplicated.bam	SampleEf_ROC2_ST10_25ng_LIB4.position_deduplicated.bam
ROC	SAMN16786375	SampleEf_ROC2_ST20_10ng_LIB1	paired	NextSeq 500		bam	SampleEf_ROC2_ST20_10ng_LIB1.barcode_deduplicated.bam	SampleEf_ROC2_ST20_10ng_LIB1.position_deduplicated.bam
ROC	SAMN16786375	SampleEf_ROC2_ST20_10ng_LIB2	paired	NextSeq 500		bam	SampleEf_ROC2_ST20_10ng_LIB2.barcode_deduplicated.bam	SampleEf_ROC2_ST20_10ng_LIB2.position_deduplicated.bam
ROC	SAMN16786375	SampleEf_ROC2_ST20_10ng_LIB3	paired	NextSeq 500		bam	SampleEf_ROC2_ST20_10ng_LIB3.barcode_deduplicated.bam	SampleEf_ROC2_ST20_10ng_LIB3.position_deduplicated.bam
ROC	SAMN16786375	SampleEf_ROC2_ST20_10ng_LIB4	paired	NextSeq 500		bam	SampleEf_ROC2_ST20_10ng_LIB4.barcode_deduplicated.bam	SampleEf_ROC2_ST20_10ng_LIB4.position_deduplicated.bam
ROC	SAMN16786375	SampleEf_ROC2_ST20_25ng_LIB1	paired	NextSeq 500		bam	SampleEf_ROC2_ST20_25ng_LIB1.barcode_deduplicated.bam	SampleEf_ROC2_ST20_25ng_LIB1.position_deduplicated.bam
ROC	SAMN16786375	SampleEf_ROC2_ST20_25ng_LIB2	paired	NextSeq 500		bam	SampleEf_ROC2_ST20_25ng_LIB2.barcode_deduplicated.bam	SampleEf_ROC2_ST20_25ng_LIB2.position_deduplicated.bam
ROC	SAMN16786375	SampleEf_ROC2_ST20_25ng_LIB3	paired	NextSeq 500		bam	SampleEf_ROC2_ST20_25ng_LIB3.barcode_deduplicated.bam	SampleEf_ROC2_ST20_25ng_LIB3.position_deduplicated.bam
ROC	SAMN16786375	SampleEf_ROC2_ST20_25ng_LIB4	paired	NextSeq 500		bam	SampleEf_ROC2_ST20_25ng_LIB4.barcode_deduplicated.bam	SampleEf_ROC2_ST20_25ng_LIB4.position_deduplicated.bam
ROC	SAMN16786375	SampleEf_ROC2_ST21_10ng_LIB1	paired	NextSeq 500		bam	SampleEf_ROC2_ST21_10ng_LIB1.barcode_deduplicated.bam	SampleEf_ROC2_ST21_10ng_LIB1.position_deduplicated.bam
ROC	SAMN16786375	SampleEf_ROC2_ST21_10ng_LIB2	paired	NextSeq 500		bam	SampleEf_ROC2_ST21_10ng_LIB2.barcode_deduplicated.bam	SampleEf_ROC2_ST21_10ng_LIB2.position_deduplicated.bam
ROC	SAMN16786375	SampleEf_ROC2_ST21_10ng_LIB3	paired	NextSeq 500		bam	SampleEf_ROC2_ST21_10ng_LIB3.barcode_deduplicated.bam	SampleEf_ROC2_ST21_10ng_LIB3.position_deduplicated.bam
ROC	SAMN16786375	SampleEf_ROC2_ST21_10ng_LIB4	paired	NextSeq 500		bam	SampleEf_ROC2_ST21_10ng_LIB4.barcode_deduplicated.bam	SampleEf_ROC2_ST21_10ng_LIB4.position_deduplicated.bam
ROC	SAMN16786375	SampleEf_ROC2_ST21_25ng_LIB1	paired	NextSeq 500		bam	SampleEf_ROC2_ST21_25ng_LIB1.barcode_deduplicated.bam	SampleEf_ROC2_ST21_25ng_LIB1.position_deduplicated.bam
ROC	SAMN16786375	SampleEf_ROC2_ST21_25ng_LIB2	paired	NextSeq 500		bam	SampleEf_ROC2_ST21_25ng_LIB2.barcode_deduplicated.bam	SampleEf_ROC2_ST21_25ng_LIB2.position_deduplicated.bam
ROC	SAMN16786375	SampleEf_ROC2_ST21_25ng_LIB3	paired	NextSeq 500		bam	SampleEf_ROC2_ST21_25ng_LIB3.barcode_deduplicated.bam	SampleEf_ROC2_ST21_25ng_LIB3.position_deduplicated.bam
ROC	SAMN16786375	SampleEf_ROC2_ST21_25ng_LIB4	paired	NextSeq 500		bam	SampleEf_ROC2_ST21_25ng_LIB4.barcode_deduplicated.bam	SampleEf_ROC2_ST21_25ng_LIB4.position_deduplicated.bam
ROC	SAMN17005115	SampleEfIS_ROC2_ST10_50ng_LIB1	paired	NextSeq 500		bam	SampleEfIS_ROC2_ST10_50ng_LIB1.barcode_deduplicated.bam	SampleEfIS_ROC2_ST10_50ng_LIB1.position_deduplicated.bam
ROC	SAMN17005115	SampleEfIS_ROC2_ST10_50ng_LIB2	paired	NextSeq 500		bam	SampleEfIS_ROC2_ST10_50ng_LIB2.barcode_deduplicated.bam	SampleEfIS_ROC2_ST10_50ng_LIB2.position_deduplicated.bam
ROC	SAMN17005115	SampleEfIS_ROC2_ST10_50ng_LIB3	paired	NextSeq 500		bam	SampleEfIS_ROC2_ST10_50ng_LIB3.barcode_deduplicated.bam	SampleEfIS_ROC2_ST10_50ng_LIB3.position_deduplicated.bam
ROC	SAMN17005115	SampleEfIS_ROC2_ST10_50ng_LIB4	paired	NextSeq 500		bam	SampleEfIS_ROC2_ST10_50ng_LIB4.barcode_deduplicated.bam	SampleEfIS_ROC2_ST10_50ng_LIB4.position_deduplicated.bam
ROC	SAMN17005115	SampleEfIS_ROC2_ST20_50ng_LIB1	paired	NextSeq 500		bam	SampleEfIS_ROC2_ST20_50ng_LIB1.barcode_deduplicated.bam	SampleEfIS_ROC2_ST20_50ng_LIB1.position_deduplicated.bam
ROC	SAMN17005115	SampleEfIS_ROC2_ST20_50ng_LIB2	paired	NextSeq 500		bam	SampleEfIS_ROC2_ST20_50ng_LIB2.barcode_deduplicated.bam	SampleEfIS_ROC2_ST20_50ng_LIB2.position_deduplicated.bam
ROC	SAMN17005115	SampleEfIS_ROC2_ST20_50ng_LIB3	paired	NextSeq 500		bam	SampleEfIS_ROC2_ST20_50ng_LIB3.barcode_deduplicated.bam	SampleEfIS_ROC2_ST20_50ng_LIB3.position_deduplicated.bam
ROC	SAMN17005115	SampleEfIS_ROC2_ST20_50ng_LIB4	paired	NextSeq 500		bam	SampleEfIS_ROC2_ST20_50ng_LIB4.barcode_deduplicated.bam	SampleEfIS_ROC2_ST20_50ng_LIB4.position_deduplicated.bam
ROC	SAMN17005115	SampleEfIS_ROC2_ST21_50ng_LIB1	paired	NextSeq 500		bam	SampleEfIS_ROC2_ST21_50ng_LIB1.barcode_deduplicated.bam	SampleEfIS_ROC2_ST21_50ng_LIB1.position_deduplicated.bam
ROC	SAMN17005115	SampleEfIS_ROC2_ST21_50ng_LIB2	paired	NextSeq 500		bam	SampleEfIS_ROC2_ST21_50ng_LIB2.barcode_deduplicated.bam	SampleEfIS_ROC2_ST21_50ng_LIB2.position_deduplicated.bam
ROC	SAMN17005115	SampleEfIS_ROC2_ST21_50ng_LIB3	paired	NextSeq 500		bam	SampleEfIS_ROC2_ST21_50ng_LIB3.barcode_deduplicated.bam	SampleEfIS_ROC2_ST21_50ng_LIB3.position_deduplicated.bam
ROC	SAMN17005115	SampleEfIS_ROC2_ST21_50ng_LIB4	paired	NextSeq 500		bam	SampleEfIS_ROC2_ST21_50ng_LIB4.barcode_deduplicated.bam	SampleEfIS_ROC2_ST21_50ng_LIB4.position_deduplicated.bam
ROC	SAMN17005116	SampleEp_ROC2_ST10_25ng_LIB1	paired	NextSeq 500		bam	SampleEp_ROC2_ST10_25ng_LIB1.barcode_deduplicated.bam	SampleEp_ROC2_ST10_25ng_LIB1.position_deduplicated.bam
ROC	SAMN17005116	SampleEp_ROC2_ST10_25ng_LIB2	paired	NextSeq 500		bam	SampleEp_ROC2_ST10_25ng_LIB2.barcode_deduplicated.bam	SampleEp_ROC2_ST10_25ng_LIB2.position_deduplicated.bam
ROC	SAMN17005116	SampleEp_ROC2_ST10_25ng_LIB3	paired	NextSeq 500		bam	SampleEp_ROC2_ST10_25ng_LIB3.barcode_deduplicated.bam	SampleEp_ROC2_ST10_25ng_LIB3.position_deduplicated.bam
ROC	SAMN17005116	SampleEp_ROC2_ST10_25ng_LIB4	paired	NextSeq 500		bam	SampleEp_ROC2_ST10_25ng_LIB4.barcode_deduplicated.bam	SampleEp_ROC2_ST10_25ng_LIB4.position_deduplicated.bam
ROC	SAMN17005116	SampleEp_ROC2_ST20_25ng_LIB1	paired	NextSeq 500		bam	SampleEp_ROC2_ST20_25ng_LIB1.barcode_deduplicated.bam	SampleEp_ROC2_ST20_25ng_LIB1.position_deduplicated.bam
ROC	SAMN17005116	SampleEp_ROC2_ST20_25ng_LIB2	paired	NextSeq 500		bam	SampleEp_ROC2_ST20_25ng_LIB2.barcode_deduplicated.bam	SampleEp_ROC2_ST20_25ng_LIB2.position_deduplicated.bam
ROC	SAMN17005116	SampleEp_ROC2_ST20_25ng_LIB3	paired	NextSeq 500		bam	SampleEp_ROC2_ST20_25ng_LIB3.barcode_deduplicated.bam	SampleEp_ROC2_ST20_25ng_LIB3.position_deduplicated.bam
ROC	SAMN17005116	SampleEp_ROC2_ST20_25ng_LIB4	paired	NextSeq 500		bam	SampleEp_ROC2_ST20_25ng_LIB4.barcode_deduplicated.bam	SampleEp_ROC2_ST20_25ng_LIB4.position_deduplicated.bam
ROC	SAMN17005116	SampleEp_ROC2_ST21_25ng_LIB1	paired	NextSeq 500		bam	SampleEp_ROC2_ST21_25ng_LIB1.barcode_deduplicated.bam	SampleEp_ROC2_ST21_25ng_LIB1.position_deduplicated.bam
ROC	SAMN17005116	SampleEp_ROC2_ST21_25ng_LIB2	paired	NextSeq 500		bam	SampleEp_ROC2_ST21_25ng_LIB2.barcode_deduplicated.bam	SampleEp_ROC2_ST21_25ng_LIB2.position_deduplicated.bam
ROC	SAMN17005116	SampleEp_ROC2_ST21_25ng_LIB3	paired	NextSeq 500		bam	SampleEp_ROC2_ST21_25ng_LIB3.barcode_deduplicated.bam	SampleEp_ROC2_ST21_25ng_LIB3.position_deduplicated.bam
ROC	SAMN17005116	SampleEp_ROC2_ST21_25ng_LIB4	paired	NextSeq 500		bam	SampleEp_ROC2_ST21_25ng_LIB4.barcode_deduplicated.bam	SampleEp_ROC2_ST21_25ng_LIB4.position_deduplicated.bam
TFS	SAMN17007051	SampleAC01_TFS2_ST22_50ng_LIB1	single	Ion Torrent S5 XL	hg19/GRCh37	bam	SampleAC01_TFS2_ST22_50ng_LIB1.bam	
TFS	SAMN17007051	SampleAC01_TFS2_ST22_50ng_LIB2	single	Ion Torrent S5 XL	hg19/GRCh37	bam	SampleAC01_TFS2_ST22_50ng_LIB2.bam	
TFS	SAMN17007051	SampleAC01_TFS2_ST22_50ng_LIB3	single	Ion Torrent S5 XL	hg19/GRCh37	bam	SampleAC01_TFS2_ST22_50ng_LIB3.bam	
TFS	SAMN17007051	SampleAC01_TFS2_ST22_50ng_LIB4	single	Ion Torrent S5 XL	hg19/GRCh37	bam	SampleAC01_TFS2_ST22_50ng_LIB4.bam	
TFS	SAMN17007051	SampleAC01_TFS2_ST23_50ng_LIB1	single	Ion Torrent S5 XL	hg19/GRCh37	bam	SampleAC01_TFS2_ST23_50ng_LIB1.bam	
TFS	SAMN17007051	SampleAC01_TFS2_ST23_50ng_LIB2	single	Ion Torrent S5 XL	hg19/GRCh37	bam	SampleAC01_TFS2_ST23_50ng_LIB2.bam	
TFS	SAMN17007051	SampleAC01_TFS2_ST23_50ng_LIB3	single	Ion Torrent S5 XL	hg19/GRCh37	bam	SampleAC01_TFS2_ST23_50ng_LIB3.bam	
TFS	SAMN17007051	SampleAC01_TFS2_ST23_50ng_LIB4	single	Ion Torrent S5 XL	hg19/GRCh37	bam	SampleAC01_TFS2_ST23_50ng_LIB4.bam	
TFS	SAMN17007051	SampleAC01_TFS2_ST24_50ng_LIB1	single	Ion Torrent S5 XL	hg19/GRCh37	bam	SampleAC01_TFS2_ST24_50ng_LIB1.bam	
TFS	SAMN17007051	SampleAC01_TFS2_ST24_50ng_LIB2	single	Ion Torrent S5 XL	hg19/GRCh37	bam	SampleAC01_TFS2_ST24_50ng_LIB2.bam	
TFS	SAMN17007051	SampleAC01_TFS2_ST24_50ng_LIB3	single	Ion Torrent S5 XL	hg19/GRCh37	bam	SampleAC01_TFS2_ST24_50ng_LIB3.bam	
TFS	SAMN17007051	SampleAC01_TFS2_ST24_50ng_LIB4	single	Ion Torrent S5 XL	hg19/GRCh37	bam	SampleAC01_TFS2_ST24_50ng_LIB4.bam	
TFS	SAMN16786373	SampleBf_TFS2_ST22_25ng_LIB1	single	Ion Torrent S5 XL	hg19/GRCh37	bam	SampleBf_TFS2_ST22_25ng_LIB1.bam	
TFS	SAMN16786373	SampleBf_TFS2_ST22_25ng_LIB2	single	Ion Torrent S5 XL	hg19/GRCh37	bam	SampleBf_TFS2_ST22_25ng_LIB2.bam	
TFS	SAMN16786373	SampleBf_TFS2_ST22_25ng_LIB3	single	Ion Torrent S5 XL	hg19/GRCh37	bam	SampleBf_TFS2_ST22_25ng_LIB3.bam	
TFS	SAMN16786373	SampleBf_TFS2_ST22_25ng_LIB4	single	Ion Torrent S5 XL	hg19/GRCh37	bam	SampleBf_TFS2_ST22_25ng_LIB4.bam	
TFS	SAMN16786373	SampleBf_TFS2_ST23_25ng_LIB1	single	Ion Torrent S5 XL	hg19/GRCh37	bam	SampleBf_TFS2_ST23_25ng_LIB1.bam	
TFS	SAMN16786373	SampleBf_TFS2_ST23_25ng_LIB2	single	Ion Torrent S5 XL	hg19/GRCh37	bam	SampleBf_TFS2_ST23_25ng_LIB2.bam	
TFS	SAMN16786373	SampleBf_TFS2_ST23_25ng_LIB3	single	Ion Torrent S5 XL	hg19/GRCh37	bam	SampleBf_TFS2_ST23_25ng_LIB3.bam	
TFS	SAMN16786373	SampleBf_TFS2_ST23_25ng_LIB4	single	Ion Torrent S5 XL	hg19/GRCh37	bam	SampleBf_TFS2_ST23_25ng_LIB4.bam	
TFS	SAMN16786373	SampleBf_TFS2_ST24_25ng_LIB1	single	Ion Torrent S5 XL	hg19/GRCh37	bam	SampleBf_TFS2_ST24_25ng_LIB1.bam	
TFS	SAMN16786373	SampleBf_TFS2_ST24_25ng_LIB2	single	Ion Torrent S5 XL	hg19/GRCh37	bam	SampleBf_TFS2_ST24_25ng_LIB2.bam	
TFS	SAMN16786373	SampleBf_TFS2_ST24_25ng_LIB3	single	Ion Torrent S5 XL	hg19/GRCh37	bam	SampleBf_TFS2_ST24_25ng_LIB3.bam	
TFS	SAMN16786373	SampleBf_TFS2_ST24_25ng_LIB4	single	Ion Torrent S5 XL	hg19/GRCh37	bam	SampleBf_TFS2_ST24_25ng_LIB4.bam	
TFS	SAMN16786374	SampleDf_TFS2_ST22_25ng_LIB1	single	Ion Torrent S5 XL	hg19/GRCh37	bam	SampleDf_TFS2_ST22_25ng_LIB1.bam	
TFS	SAMN16786374	SampleDf_TFS2_ST22_25ng_LIB2	single	Ion Torrent S5 XL	hg19/GRCh37	bam	SampleDf_TFS2_ST22_25ng_LIB2.bam	
TFS	SAMN16786374	SampleDf_TFS2_ST22_25ng_LIB3	single	Ion Torrent S5 XL	hg19/GRCh37	bam	SampleDf_TFS2_ST22_25ng_LIB3.bam	
TFS	SAMN16786374	SampleDf_TFS2_ST22_25ng_LIB4	single	Ion Torrent S5 XL	hg19/GRCh37	bam	SampleDf_TFS2_ST22_25ng_LIB4.bam	
TFS	SAMN16786374	SampleDf_TFS2_ST23_25ng_LIB1	single	Ion Torrent S5 XL	hg19/GRCh37	bam	SampleDf_TFS2_ST23_25ng_LIB1.bam	
TFS	SAMN16786374	SampleDf_TFS2_ST23_25ng_LIB2	single	Ion Torrent S5 XL	hg19/GRCh37	bam	SampleDf_TFS2_ST23_25ng_LIB2.bam	
TFS	SAMN16786374	SampleDf_TFS2_ST23_25ng_LIB3	single	Ion Torrent S5 XL	hg19/GRCh37	bam	SampleDf_TFS2_ST23_25ng_LIB3.bam	
TFS	SAMN16786374	SampleDf_TFS2_ST23_25ng_LIB4	single	Ion Torrent S5 XL	hg19/GRCh37	bam	SampleDf_TFS2_ST23_25ng_LIB4.bam	
TFS	SAMN16786374	SampleDf_TFS2_ST24_25ng_LIB1	single	Ion Torrent S5 XL	hg19/GRCh37	bam	SampleDf_TFS2_ST24_25ng_LIB1.bam	
TFS	SAMN16786374	SampleDf_TFS2_ST24_25ng_LIB2	single	Ion Torrent S5 XL	hg19/GRCh37	bam	SampleDf_TFS2_ST24_25ng_LIB2.bam	
TFS	SAMN16786374	SampleDf_TFS2_ST24_25ng_LIB3	single	Ion Torrent S5 XL	hg19/GRCh37	bam	SampleDf_TFS2_ST24_25ng_LIB3.bam	
TFS	SAMN16786374	SampleDf_TFS2_ST24_25ng_LIB4	single	Ion Torrent S5 XL	hg19/GRCh37	bam	SampleDf_TFS2_ST24_25ng_LIB4.bam	
TFS	SAMN16786375	SampleEf_TFS2_ST22_10ng_LIB1	single	Ion Torrent S5 XL	hg19/GRCh37	bam	SampleEf_TFS2_ST22_10ng_LIB1.bam	
TFS	SAMN16786375	SampleEf_TFS2_ST22_10ng_LIB2	single	Ion Torrent S5 XL	hg19/GRCh37	bam	SampleEf_TFS2_ST22_10ng_LIB2.bam	
TFS	SAMN16786375	SampleEf_TFS2_ST22_10ng_LIB3	single	Ion Torrent S5 XL	hg19/GRCh37	bam	SampleEf_TFS2_ST22_10ng_LIB3.bam	
TFS	SAMN16786375	SampleEf_TFS2_ST22_10ng_LIB4	single	Ion Torrent S5 XL	hg19/GRCh37	bam	SampleEf_TFS2_ST22_10ng_LIB4.bam	
TFS	SAMN16786375	SampleEf_TFS2_ST22_25ng_LIB1	single	Ion Torrent S5 XL	hg19/GRCh37	bam	SampleEf_TFS2_ST22_25ng_LIB1.bam	
TFS	SAMN16786375	SampleEf_TFS2_ST22_25ng_LIB2	single	Ion Torrent S5 XL	hg19/GRCh37	bam	SampleEf_TFS2_ST22_25ng_LIB2.bam	
TFS	SAMN16786375	SampleEf_TFS2_ST22_25ng_LIB3	single	Ion Torrent S5 XL	hg19/GRCh37	bam	SampleEf_TFS2_ST22_25ng_LIB3.bam	
TFS	SAMN16786375	SampleEf_TFS2_ST22_25ng_LIB4	single	Ion Torrent S5 XL	hg19/GRCh37	bam	SampleEf_TFS2_ST22_25ng_LIB4.bam	
TFS	SAMN16786375	SampleEf_TFS2_ST23_10ng_LIB1	single	Ion Torrent S5 XL	hg19/GRCh37	bam	SampleEf_TFS2_ST23_10ng_LIB1.bam	
TFS	SAMN16786375	SampleEf_TFS2_ST23_10ng_LIB2	single	Ion Torrent S5 XL	hg19/GRCh37	bam	SampleEf_TFS2_ST23_10ng_LIB2.bam	
TFS	SAMN16786375	SampleEf_TFS2_ST23_10ng_LIB3	single	Ion Torrent S5 XL	hg19/GRCh37	bam	SampleEf_TFS2_ST23_10ng_LIB3.bam	
TFS	SAMN16786375	SampleEf_TFS2_ST23_10ng_LIB4	single	Ion Torrent S5 XL	hg19/GRCh37	bam	SampleEf_TFS2_ST23_10ng_LIB4.bam	
TFS	SAMN16786375	SampleEf_TFS2_ST23_25ng_LIB1	single	Ion Torrent S5 XL	hg19/GRCh37	bam	SampleEf_TFS2_ST23_25ng_LIB1.bam	
TFS	SAMN16786375	SampleEf_TFS2_ST23_25ng_LIB2	single	Ion Torrent S5 XL	hg19/GRCh37	bam	SampleEf_TFS2_ST23_25ng_LIB2.bam	
TFS	SAMN16786375	SampleEf_TFS2_ST23_25ng_LIB3	single	Ion Torrent S5 XL	hg19/GRCh37	bam	SampleEf_TFS2_ST23_25ng_LIB3.bam	
TFS	SAMN16786375	SampleEf_TFS2_ST23_25ng_LIB4	single	Ion Torrent S5 XL	hg19/GRCh37	bam	SampleEf_TFS2_ST23_25ng_LIB4.bam	
TFS	SAMN16786375	SampleEf_TFS2_ST24_10ng_LIB1	single	Ion Torrent S5 XL	hg19/GRCh37	bam	SampleEf_TFS2_ST24_10ng_LIB1.bam	
TFS	SAMN16786375	SampleEf_TFS2_ST24_10ng_LIB2	single	Ion Torrent S5 XL	hg19/GRCh37	bam	SampleEf_TFS2_ST24_10ng_LIB2.bam	
TFS	SAMN16786375	SampleEf_TFS2_ST24_10ng_LIB3	single	Ion Torrent S5 XL	hg19/GRCh37	bam	SampleEf_TFS2_ST24_10ng_LIB3.bam	
TFS	SAMN16786375	SampleEf_TFS2_ST24_10ng_LIB4	single	Ion Torrent S5 XL	hg19/GRCh37	bam	SampleEf_TFS2_ST24_10ng_LIB4.bam	
TFS	SAMN16786375	SampleEf_TFS2_ST24_25ng_LIB1	single	Ion Torrent S5 XL	hg19/GRCh37	bam	SampleEf_TFS2_ST24_25ng_LIB1.bam	
TFS	SAMN16786375	SampleEf_TFS2_ST24_25ng_LIB2	single	Ion Torrent S5 XL	hg19/GRCh37	bam	SampleEf_TFS2_ST24_25ng_LIB2.bam	
TFS	SAMN16786375	SampleEf_TFS2_ST24_25ng_LIB3	single	Ion Torrent S5 XL	hg19/GRCh37	bam	SampleEf_TFS2_ST24_25ng_LIB3.bam	
TFS	SAMN16786375	SampleEf_TFS2_ST24_25ng_LIB4	single	Ion Torrent S5 XL	hg19/GRCh37	bam	SampleEf_TFS2_ST24_25ng_LIB4.bam	
TFS	SAMN17005115	SampleEfIS_TFS2_ST22_50ng_LIB1	single	Ion Torrent S5 XL	hg19/GRCh37	bam	SampleEfIS_TFS2_ST22_50ng_LIB1.bam	
TFS	SAMN17005115	SampleEfIS_TFS2_ST22_50ng_LIB2	single	Ion Torrent S5 XL	hg19/GRCh37	bam	SampleEfIS_TFS2_ST22_50ng_LIB2.bam	
TFS	SAMN17005115	SampleEfIS_TFS2_ST22_50ng_LIB3	single	Ion Torrent S5 XL	hg19/GRCh37	bam	SampleEfIS_TFS2_ST22_50ng_LIB3.bam	
TFS	SAMN17005115	SampleEfIS_TFS2_ST22_50ng_LIB4	single	Ion Torrent S5 XL	hg19/GRCh37	bam	SampleEfIS_TFS2_ST22_50ng_LIB4.bam	
TFS	SAMN17005115	SampleEfIS_TFS2_ST23_50ng_LIB1	single	Ion Torrent S5 XL	hg19/GRCh37	bam	SampleEfIS_TFS2_ST23_50ng_LIB1.bam	
TFS	SAMN17005115	SampleEfIS_TFS2_ST23_50ng_LIB2	single	Ion Torrent S5 XL	hg19/GRCh37	bam	SampleEfIS_TFS2_ST23_50ng_LIB2.bam	
TFS	SAMN17005115	SampleEfIS_TFS2_ST23_50ng_LIB3	single	Ion Torrent S5 XL	hg19/GRCh37	bam	SampleEfIS_TFS2_ST23_50ng_LIB3.bam	
TFS	SAMN17005115	SampleEfIS_TFS2_ST23_50ng_LIB4	single	Ion Torrent S5 XL	hg19/GRCh37	bam	SampleEfIS_TFS2_ST23_50ng_LIB4.bam	
TFS	SAMN17005115	SampleEfIS_TFS2_ST24_50ng_LIB1	single	Ion Torrent S5 XL	hg19/GRCh37	bam	SampleEfIS_TFS2_ST24_50ng_LIB1.bam	
TFS	SAMN17005115	SampleEfIS_TFS2_ST24_50ng_LIB2	single	Ion Torrent S5 XL	hg19/GRCh37	bam	SampleEfIS_TFS2_ST24_50ng_LIB2.bam	
TFS	SAMN17005115	SampleEfIS_TFS2_ST24_50ng_LIB3	single	Ion Torrent S5 XL	hg19/GRCh37	bam	SampleEfIS_TFS2_ST24_50ng_LIB3.bam	
TFS	SAMN17005115	SampleEfIS_TFS2_ST24_50ng_LIB4	single	Ion Torrent S5 XL	hg19/GRCh37	bam	SampleEfIS_TFS2_ST24_50ng_LIB4.bam	
TFS	SAMN17005116	SampleEp_TFS2_ST22_25ng_LIB1	single	Ion Torrent S5 XL	hg19/GRCh37	bam	SampleEp_TFS2_ST22_25ng_LIB1.bam	
TFS	SAMN17005116	SampleEp_TFS2_ST22_25ng_LIB2	single	Ion Torrent S5 XL	hg19/GRCh37	bam	SampleEp_TFS2_ST22_25ng_LIB2.bam	
TFS	SAMN17005116	SampleEp_TFS2_ST22_25ng_LIB3	single	Ion Torrent S5 XL	hg19/GRCh37	bam	SampleEp_TFS2_ST22_25ng_LIB3.bam	
TFS	SAMN17005116	SampleEp_TFS2_ST22_25ng_LIB4	single	Ion Torrent S5 XL	hg19/GRCh37	bam	SampleEp_TFS2_ST22_25ng_LIB4.bam	
TFS	SAMN17005116	SampleEp_TFS2_ST23_25ng_LIB1	single	Ion Torrent S5 XL	hg19/GRCh37	bam	SampleEp_TFS2_ST23_25ng_LIB1.bam	
TFS	SAMN17005116	SampleEp_TFS2_ST23_25ng_LIB2	single	Ion Torrent S5 XL	hg19/GRCh37	bam	SampleEp_TFS2_ST23_25ng_LIB2.bam	
TFS	SAMN17005116	SampleEp_TFS2_ST23_25ng_LIB3	single	Ion Torrent S5 XL	hg19/GRCh37	bam	SampleEp_TFS2_ST23_25ng_LIB3.bam	
TFS	SAMN17005116	SampleEp_TFS2_ST23_25ng_LIB4	single	Ion Torrent S5 XL	hg19/GRCh37	bam	SampleEp_TFS2_ST23_25ng_LIB4.bam	
TFS	SAMN17005116	SampleEp_TFS2_ST24_25ng_LIB1	single	Ion Torrent S5 XL	hg19/GRCh37	bam	SampleEp_TFS2_ST24_25ng_LIB1.bam	
TFS	SAMN17005116	SampleEp_TFS2_ST24_25ng_LIB2	single	Ion Torrent S5 XL	hg19/GRCh37	bam	SampleEp_TFS2_ST24_25ng_LIB2.bam	
TFS	SAMN17005116	SampleEp_TFS2_ST24_25ng_LIB3	single	Ion Torrent S5 XL	hg19/GRCh37	bam	SampleEp_TFS2_ST24_25ng_LIB3.bam	
TFS	SAMN17005116	SampleEp_TFS2_ST24_25ng_LIB4	single	Ion Torrent S5 XL	hg19/GRCh37	bam	SampleEp_TFS2_ST24_25ng_LIB4.bam	
TFS	SAMN17007052	SampleFf_TFS2_ST22_100ng_LIB1	single	Ion Torrent S5 XL	hg19/GRCh37	bam	SampleFf_TFS2_ST22_100ng_LIB1.bam	
TFS	SAMN17007052	SampleFf_TFS2_ST22_100ng_LIB2	single	Ion Torrent S5 XL	hg19/GRCh37	bam	SampleFf_TFS2_ST22_100ng_LIB2.bam	
TFS	SAMN17007052	SampleFf_TFS2_ST22_100ng_LIB3	single	Ion Torrent S5 XL	hg19/GRCh37	bam	SampleFf_TFS2_ST22_100ng_LIB3.bam	
TFS	SAMN17007052	SampleFf_TFS2_ST22_100ng_LIB4	single	Ion Torrent S5 XL	hg19/GRCh37	bam	SampleFf_TFS2_ST22_100ng_LIB4.bam	
TFS	SAMN17007052	SampleFf_TFS2_ST23_100ng_LIB1	single	Ion Torrent S5 XL	hg19/GRCh37	bam	SampleFf_TFS2_ST23_100ng_LIB1.bam	
TFS	SAMN17007052	SampleFf_TFS2_ST23_100ng_LIB2	single	Ion Torrent S5 XL	hg19/GRCh37	bam	SampleFf_TFS2_ST23_100ng_LIB2.bam	
TFS	SAMN17007052	SampleFf_TFS2_ST23_100ng_LIB3	single	Ion Torrent S5 XL	hg19/GRCh37	bam	SampleFf_TFS2_ST23_100ng_LIB3.bam	
TFS	SAMN17007052	SampleFf_TFS2_ST23_100ng_LIB4	single	Ion Torrent S5 XL	hg19/GRCh37	bam	SampleFf_TFS2_ST23_100ng_LIB4.bam	
TFS	SAMN17007052	SampleFf_TFS2_ST24_100ng_LIB2	single	Ion Torrent S5 XL	hg19/GRCh37	bam	SampleFf_TFS2_ST24_100ng_LIB2.bam	
TFS	SAMN17007052	SampleFf_TFS2_ST24_100ng_LIB3	single	Ion Torrent S5 XL	hg19/GRCh37	bam	SampleFf_TFS2_ST24_100ng_LIB3.bam	
TFS	SAMN17007052	SampleFf_TFS2_ST24_100ng_LIB4	single	Ion Torrent S5 XL	hg19/GRCh37	bam	SampleFf_TFS2_ST24_100ng_LIB4.bam	
